# SARS-CoV-2 Mpro inhibitor ensitrelvir: asymmetrical cross-resistance with nirmatrelvir and emerging resistance hotspots

**DOI:** 10.1080/22221751.2025.2552716

**Published:** 2025-10-14

**Authors:** Yuyong Zhou, Karen A. Gammeltoft, Helena D. Tjørnelund-Sjursen, Line A. Ryberg, Anna Offersgaard, Anna Czarnota, Zhe Duan, Long V. Pham, Ulrik Fahnøe, Günther H.J. Peters, Santseharay Ramirez, Jens Bukh, Judith M. Gottwein

**Affiliations:** a Copenhagen Hepatitis C Program (CO-HEP), Department of Infectious Diseases, Copenhagen University Hospital–Hvidovre, Hvidovre, Denmark; b Copenhagen Hepatitis C Program (CO-HEP), Department of Immunology and Microbiology, Faculty of Health and Medical Sciences, University of Copenhagen, Copenhagen, Denmark; c Department of Chemistry, Technical University of Denmark, Kongens Lyngby, Denmark

**Keywords:** Antiviral resistance, SARS-CoV-2, protease inhibitor, ensitrelvir, nirmatrelvir

## Abstract

SARS-CoV-2 main protease (Mpro) inhibitors are the first-line COVID-19 treatment. Nirmatrelvir is used worldwide, while ensitrelvir, licensed in Japan and Singapore, has received FDA fast-track designation. To facilitate population monitoring for viral resistance and guide next-generation inhibitor design, we investigated SARS-CoV-2 resistance and cross-resistance to ensitrelvir and nirmatrelvir. SARS-CoV-2 escape variants with high fitness and high ensitrelvir resistance were selected under clinically relevant concentrations in infectious cell culture assays. Using infectious cell culture, replicon, and Mpro assays, reverse genetics revealed synergistic combinations of resistance-associated substitutions (RAS), specifically M49L + S144A and M49L + S144A + T169I, that conferred high resistance with a low fitness cost. Molecular dynamics simulations confirmed that M49L + S144A or M49L + S144A + T169I weakened ensitrelvir-Mpro binding. M49L + S144A and M49L + S144A + T169I exhibited a lower fitness cost and conferred higher resistance than the previously identified ensitrelvir RAS M49L + E166A. Cross-resistance between these ensitrelvir RAS and previously described nirmatrelvir RAS L50F + E166V was asymmetrical, with nirmatrelvir RAS showing greater resistance to ensitrelvir than vice versa. Amino acid changes at Mpro-position 166, an emerging resistance hotspot with natural variation, had differential impacts on viral fitness and Mpro inhibitor resistance in infectious cell culture assays. The most frequently naturally occurring substitution, E166Q, did not confer significant resistance to either ensitrelvir or nirmatrelvir. However, the second most frequent substitution, E166H, conferred high resistance to nirmatrelvir, but not to ensitrelvir. This comparative resistance analysis can inform COVID-19 treatment strategies and contribute to pandemic preparedness.

## Introduction

The coronavirus disease 2019 (COVID-19) pandemic remains the most devastating viral outbreak in modern history, profoundly affecting human societies worldwide. Hospital and mortality burden of COVID-19 remains high, even in societies with robust immunity, whether from vaccination or natural infection [1].

The first-line COVID-19 treatment options are oral inhibitors of the main protease (Mpro) of severe acute respiratory syndrome coronavirus 2 (SARS-CoV-2). These compounds are particularly effective in preventing hospitalizations and deaths of high-risk individuals. Nirmatrelvir (the active compound in Paxlovid) has been approved by the Food and Drug Administration (FDA) and the European Medicines Agency and is widely used worldwide [2,3]. Ensitrelvir (Xocova), with a different chemical structure, is licensed in Japan and Singapore. It received fast-track designation by the FDA and is currently undergoing Phase 3 clinical trials involving patients worldwide, including patients in the U.S. and Europe [4–6]. Simnotrelvir (Xiannuoxin), with a similar chemical structure as nirmatrelvir, is licensed in China [7,8]. Additionally, several other small molecule Mpro inhibitors are being developed [9–16]. In comparison, the intravenous polymerase inhibitor remdesivir is used less frequently, and mostly to treat severe COVID-19 in hospitalized patients [17,18]. In contrast, the nucleotide analogue molnupiravir and monoclonal antibodies are no longer primary treatment options due to their limited efficacy and the emergence of resistant viral variants, respectively [19–21].

The efficacy of currently highly effective Mpro inhibitors might be threatened by viral resistance development, and identifying the genetic correlates of viral resistance is essential for population monitoring and for guiding treatment strategies. Similarly, evaluating the fitness of resistant variants is important to predict the likelihood of their occurrence and persistence. We previously identified nirmatrelvir resistance-associated substitutions (RAS) in SARS-CoV-2 in vitro [22]. These RAS were confirmed by others and detected in treated COVID-19 patients, highlighting the relevance of the applied SARS-CoV-2 in vitro systems for translational studies [23–30]. Here, we characterized genetic correlates of ensitrelvir resistance in established in vitro systems, including a highly reproducible reverse-genetics system [22,31–33], applying physiological inhibitor concentrations. We further investigated cross-resistance between ensitrelvir and nirmatrelvir. Finally, we aimed to evaluate the impact of genetic variation at Mpro-position 166.

## Materials and methods

### Cells, previously generated virus stocks and inhibitors

VeroE6 monkey kidney cells (generously provided by J. Dubuisson) and A549-hACE2 human lung cells (InvivoGen) were cultured as described [22,31,34,35]. Cell culture flasks, plates, and chamber slides were from Thermo Fisher Scientific. Infectious cell culture experiments were carried out under biosafety conditions in accordance with Danish regulations and with permission from the Danish authorities.

Virus stocks were generated in VeroE6 cells and sequence confirmed by next-generation sequencing (NGS). The reference virus termed “original virus” (SARS-CoV-2/human/Denmark/DK-AHH1/2020, GenBank: MZ049597) was isolated from a Danish patient early in the pandemic [22,36]. The polyclonal nirmatrelvir escape virus was from a nirmatrelvir escape experiment with the original virus [22]. The recombinant E166V and L50F + E166V variants were engineered using the bacterial artificial chromosome (BAC) clone reflecting the sequence of the original virus [32] and propagated as described [22].

Ensitrelvir (Selleck Chemicals (E1131) or MedChemExpress (HY-143216A)) was dissolved in DMSO (Thermo Fisher Scientific) to a final concentration of 30.9 mM and stored at −80 °C. Nirmatrelvir (Acme Bioscience), was dissolved in DMSO to a final concentration of 40 mM and stored at −20 °C.

### Selection of ensitrelvir RAS

In two independent escape experiments, 1 × 10^6^ VeroE6 cells seeded the previous day in T25 flasks, were infected with the original SARS-CoV-2 virus at a multiplicity of infection of 0.00002. Ensitrelvir was added at 4- or 5-fold 50% effective concentration (EC50) (EC50 = 0.42 μM) directly after infection and every 2–3 days, when cells were split including seeding of replicate cultures for immunostaining of SARS-CoV-2 spike antigen. When 90% of cells were estimated to be infected based on immunostaining, the virus was passaged by inoculation of 5 µL, which were derived from a total of 4 mL cell culture supernatant from the preceding culture to 1 × 10^6^ naïve cells seeded the previous day in a T25 flask. Inhibitor concentrations were stepwise increased to up to 364-fold EC50.

Polyclonal ENS-EV1 and ENS-EV2 escape virus stocks were generated by inoculating 3 × 10^6^ VeroE6 cells, seeded the previous day in T80 flasks, with 250 µL of supernatant from passage 6 day post infection (DPI) 6 (escape 1), or passage 4 DPI 8 (escape 2), respectively. Supernatants from the peak of infection were pooled and the resulting virus stocks were stored at −80 °C.

### Immunostaining

Immunostaining was done during viral escape, transfection and passage experiments as described [22,31]. Upon cells splitting, replicate cultures were seeded in 8-well chamber slides. The following day, cells were fixed with methanol (J.T. Baker). Cells were stained using primary antibody SARS-CoV-2 spike chimeric monoclonal antibody (Sino Biological, #40150-D004) diluted 1:1000 and secondary antibody Alexa Fluor 488 goat anti-human IgG (Invitrogen, #A-11013) diluted 1:500, along with Hoechst 33342 (Invitrogen) diluted 1:1000. Fluorescence microscopy (ZEISS Axio Vert.A1) was used to evaluate the percentage of spike protein-positive cells, with categories 0%, single infected cells, and 10-90% infected cells in increments of 10%.

### Generation of recombinant SARS-CoV-2 variants

Putative ensitrelvir RAS (M49L, S144A, T169I, E166A, M49L + S144A, M49L + S144A + T169I or M49L + E166A) and specified amino acid changes at Mpro-position 166 were introduced into the BAC clone reflecting the sequence of the original AHH1 virus [32] using In-Fusion Snap Assembly master mix (Takara). Minipreps and maxipreps were generated using QIAprep Spin Miniprep Kit (QIAGEN) and HiSpeed® Plasmid Maxi Kit (QIAGEN), respectively; their complete SARS-CoV-2 sequence was confirmed by NGS.

SARS-CoV-2 in vitro RNA transcripts were generated as described [22,31–33]. 2 × 10^5^ VeroE6 cells, seeded the previous day in 12-well plates, were transfected with 5000 ng RNA transcripts and 5 μl Lipofectamine 2000 (Thermo Fisher Scientific). Cultures were monitored for cytopathogenic effects (CPE) in an inverted light microscope, assigning categories slight, moderate and massive CPE, and for the percentage of infected cells by immunostaining. Recombinants were termed “non-viable” if there was no signs of CPE and no infected cells for at least 7 days post transfection in at least 3 independent experiments.

For viable variants, first passage virus stocks were generated by inoculation of 3 × 10^6^ VeroE6 cells, seeded the previous day in T80 flasks, with 250 or 500 μL transfection supernatant from the peak of infection [22,31,32]. Supernatants from the peak of infection were pooled to generate the virus stocks.

### Serial passage of recombinant SARS-CoV-2 variants

Variants were passaged by inoculation of 1 × 10^6^ VeroE6 cells, seeded the previous day in T25 flask, with 250 µL of supernatant obtained at the peak of infection from the previous culture.

### NGS

NGS was used for primary escape cultures, passage cultures, virus stocks and plasmids. For cell culture derived SARS-CoV-2, viral RNA was extracted, purified, and subjected to RT–PCR to generate five overlapping amplicons [32,36]. The primers were specified in [32] and located outside the Mpro gene. The NEBNext Ultra II FS DNA Library Prep Kit (New England BioLabs) and the Illumina platform were used [22,37].

### Infectivity titrations

SARS-CoV-2 infectivity titres of first passage virus stocks or supernatants from day 3 post transfection were determined as described [22]. 1 × 10^4^ VeroE6 cells, seeded the previous day in 96-well clear bottom plates, were infected with 10-fold serial dilutions of supernatants in 4 replicates. Three days post infection, plates were fixed with methanol and stained with primary antibody SARS-CoV-2 spike chimeric monoclonal antibody (Sino Biological #40150-D004) at 1:5000 dilution and secondary antibody F(ab’)2-Goat anti-human IgG Fc Cross-Adsorbed Secondary Antibody, HRP (Invitrogen#A24476) at 1:2000 dilution. Infected cells were visualized with the BrightDAB kit (Immunologic, #BS04-110) and wells were scored infected or non-infected using the ImmunoSpot Series 5 UV Analyzer (CTL Europe GmbH). Infectivity titres were determined as TCID50/mL using the Reed-Muench method [38] and are means with standard errors of the means (SEM) from three independent experiments, each with 4 technical replicates. The lower limit of detection was 2 log_10_TCID50/mL.

### Competition growth assays

Competition growth assays were carried out in VeroE6 cells as described [22]. 1 × 10^6^ cells, plated the previous day in a T25 flask, were infected with the original recombinant SARS-CoV-2 and the M49L + S144A variant or with the original recombinant SARS-CoV-2 and the M49L + S144A + T169I variant at different ratios. Control cultures were infected solely with one of these viruses. Infections were conducted at a total multiplicity of infection of 0.0001, while the ratio between the two viruses varied as specified. Cell culture supernatant was collected every day and stored at −80°C, and fresh medium was added. Using supernatants collected on day 4 post-infection, at which point most of the cultured cells were estimated to be infected by CPE evaluation, the frequencies of the respective virus populations were determined by NGS based on the frequency of M49L. Frequencies are means of measurements derived from three replicate cultures.

### Antiviral assays

Antiviral assays were carried out in VeroE6 and A549-hACE2 cells, as described [22,31,34,39]. 1 × 10^4^ cells, seeded the previous day in 96-well plates, were infected with specified virus stocks and treated with serial inhibitor dilutions in 4 replicates, including non-treated infected and non-treated non-infected controls in 8 replicates. The original virus served as reference [36]. Two days post infection, plates were fixed and stained as described in “Infectivity titration”. The number of single SARS-CoV-2 spike-positive cells per well was evaluated with the ImmunoSpot Series 5 UV Analyzer [39]. Residual infectivity values (%) were calculated for each treated infected culture by comparing the number of positive cells in this culture to the mean count of positive cells in the non-treated infected cultures and are means of 4 replicates with SEM. Concentration–response curves and EC50 were determined as described in “Statistical analysis”. Cell viability at specified inhibitor concentrations was evaluated as described [22,31,34,35,39].

### Replicon assays

Replicon assays were done as described using the nLuc ΔS-E-M system, including RNA transcript generation, transfection, and luciferase activity measurement [22,32,40]. 1 × 10^5^ VeroE6 cells, seeded the previous day in 24-well plates, were transfected with 500 ng of RNA transcripts for 1 h. Following transfection, supernatants were replaced with fresh media, with specified inhibitor concentrations. Luciferase activity was assessed using the Nano-Glo Luciferase Assay System (Promega). Each condition was tested in duplicate cultures and measured with three technical replicates. Mock transfections (without RNA) were used as controls. Relative light units (RLU) were measured using the Synergy LX Multi-Mode Microplate Reader (BioTek) at 1, 24 and 48 h post transfection. Baseline (1 h) values were subtracted from subsequent measurements, and the mean of blank controls was subtracted from all measurements for each plate. For non-treated cultures, RLU were normalized to the mean RLU of the original virus. For treated cultures, RLU at 24 h were normalized to the corresponding non-treated controls.

### Expression and purification of Mpro

The SARS-CoV-2 Mpro sequence, codon-optimized for expression in *E. coli*, was synthesized by Genscript and engineered into the pET SUMO expression vector (Invitrogen) by In-Fusion cloning. Mutations were engineered by QuikChange Lightning (Agilent), and Mpro sequences of final plasmid preparations were confirmed by NGS.

Plasmids were transformed into BL21(DE3) *E. coli* (Thermo Fischer Scientific) and grown in 2xYT media (Sigma-Aldrich) containing 50 ng/μL kanamycin at 37 ⁰C, shaking at 230 revolutions per minute (RPM). Protein expression was induced by isopropyl β-d-1-thiogalactopyranoside (IPTG) at 1 mM, 18 °C, and 150 RPM. After 16–20 h, cells were pelleted by centrifugation at 4000 g for 20 min at 4 °C, the pellet was resuspended in buffer A (20 mM Tris, 300 mM NaCl, 10 mM imidazole, 1 mM DTT, pH 8.0). Cells were lysed by sonication (8 × 30 s with breaks of 30 s in between) on ice, the lysate was cleared by centrifugation at 20,000 g for 60 min at 4 °C. Mpro in the soluble fraction was purified using an ÄktaPure (Cytiva) and HisTrap HP columns (Cytiva). In brief, the lysate was filtered (0.45 µm) and loaded on the HisTrap HP column followed by washing with buffer A. Recombinant His-Sumo-Mpro fusion proteins were eluted in buffer B (20 mM Tris, 300 mM NaCl, 300 mM imidazole, 1 mM DTT, pH 8.0) using a linear gradient. To remove the His-SUMO-tags, the eluted proteins were digested over night with SUMO-protease (ThermoFischer) at 4 °C. The following day, the digested proteins were loaded on HisTrap HP columns to remove the His-SUMO tags, uncleaved His-SUMO-Mpro and the His-tagged SUMO proteases. Untagged Mpro was eluted in the flowthrough during the loading and washing of the column with buffer A. This purified Mpro in buffer A was subjected to buffer exchange with buffer C (20 mM HEPES, 120 mM NaCl, 0.4 mM EDTA, 4 mM DTT, and 20% glycerol, pH 6.5) using PD-10 desalting columns (Cytiva). Proteins were stored at –80 °C.

### Mpro enzymatic assays

Enzymatic assays were carried out as described, with minor modifications [22]. For determination of Mpro activity, 100 nM Mpro was mixed with 0-150 µM FRET substrate (Dabcyl-KTSAVLQ/SGFRKM-E(Edans)-NH2, Biopeptide), and relative fluorescence units (RFU) were recorded continuously for 1 h using the VANTAstar (BMG LabTech) with filters for excitation at 340 nm and emission at 490 nm. The recorded RFU were converted to µM cleaved FRET substrate using a standard curve. The initial velocity was calculated based on the initial linear part of the reaction. The initial velocities in µM/min were plotted against the concentration of FRET substrate to determine Km and Vmax as described in “Statistical analysis”. Relative Vmax and Km of Mpro variants were determined by normalizing to Vmax and Km of the original Mpro determined in the same experiment.

For EC50 determinations, Mpro variants at a final concentration of 100 nM were incubated for 10 min at room temperature with ensitrelvir or nirmatrelvir as specified. Reactions were initiated with the addition of 20 µM FRET substrate. RFU were recorded continuously for 1 h using the FLUOstar OPTIMA (BMG LabTech) with filters for excitation at 360 nm and emission at 505 nm. Residual initial velocity was determined by relating the initial Mpro velocities of the reactions with inhibitor to the initial velocity of the reactions without inhibitor, for each Mpro variant. Residual initial velocity was plotted against the inhibitor concentration. Concentration–response curves and EC50 were determined as described in “Statistical analysis”.

### Molecular dynamics simulations

The molecular dynamics simulations (MDS) were done as described [22] with minor modifications. In brief, the crystal structures of Mpro in complex with nsp4/nsp5 substrate peptide (Protein Data Bank (PDB) ID: 7MGS [41]) and ensitrelvir (PDB ID: 8HBK [42]) were used as input structures for the MDS. The PyMOL software (version 3.0.2, Schrödinger, LCC) was used for preparing the structures for simulation including generating Mpro dimers and introducing relevant substitutions (M49L, T169I, S144A, M49L + S144A or M49L + S144A + T169I). For the Mpro-ensitrelvir complex, the C-terminal residues 301–306 were missing in the structure, and these residues were added from another Mpro structure (PDB ID: 7JP1 [43]). The Mpro dimers were solvated in dodecahedron-shaped water boxes with a minimum distance between protein and water box edges of 12 Å. To replicate the cytosolic physiological ionic strength, Na^+^ and Cl^-^ ions were included in the models to a final concentration of 150 mM [44]. The CHARMM36m all-atom protein force field and the CHARMM TIP3P water model were used for the simulations [45,46]. A topology file for ensitrelvir was generated using the CHARMM General Force Field (CGenFF) using the CHARMM-GUI [47,48].

All MDS were carried out in GROMACS (version 2021.2) using the leap-frog algorithm and a time step of 1 fs [49,50]. The prepared systems were first energy-minimized followed by two equilibration phases of 100 ps, each in isothermal-isochoric and isothermal-isobaric ensembles, respectively. The systems were simulated in an isothermal-isobaric ensemble at 310 K and 1 bar for 200 ns. Each system was simulated in triplicate to assess the statistical significance of the simulation results. The systems were minimized individually, and the MDS were initiated with different initial atom velocities chosen randomly from a Maxwell–Boltzmann velocity distribution.

The trajectories were subsequently analysed to extract geometrical protein properties and protein–ligand interaction patterns using gmx modules in GROMACS. The root-mean-squared deviations of backbone atoms were calculated with respect to the minimized structures. The interaction energies between the Mpro dimer and either ensitrelvir or the substrate peptide were calculated as a sum of Lennard-Jones and Coulomb interaction energies. The interaction energies of the Mpro variants were compared to that of the original: Δ*E* = *E*_variant Mpro_ – *E*_original Mpro_ with negative/positive energy differences corresponding to improved/weakened binding, respectively [22].

A contact heatmap was generated based on distance calculations carried out using VMD (version 1.9.4) [51] counting Mpro residues within a maximum distance of 3 Å from ensitrelvir or the substrate peptide for all simulation frames. The frequency of these distances, *f*, correlates with interaction energies; thus, higher contact frequency indicated stronger interactions. Δ*f* = *f*_variant Mpro_ – *f*_original Mpro_ was plotted in a contact heatmap. Residues with *f* < 5% were not included in the heatmap.

### Statistical analysis

The described calculations were carried out using GraphPad Prism 10.1.2. For antiviral assays and Mpro enzymatic assays, EC50 were determined as Y = Top/(1 + 10^((LogEC50-X)×HillSlope)).

For Mpro enzymatic assays, the initial velocities were determined by simple linear regression, and Km and Vmax using the Michaelis–Menten equation V_0_ = (V_max_×[S_0_])/(K_m_ + [S_0_]).

Pearson correlation analysis was used to evaluate the correlation between EC50 determined in Mpro enzymatic assays and EC50 determined in cell-based antiviral assays.

## Results

### SARS-CoV-2 ensitrelvir escape viruses showed limited cross-resistance to nirmatrelvir

**Figure 1.**
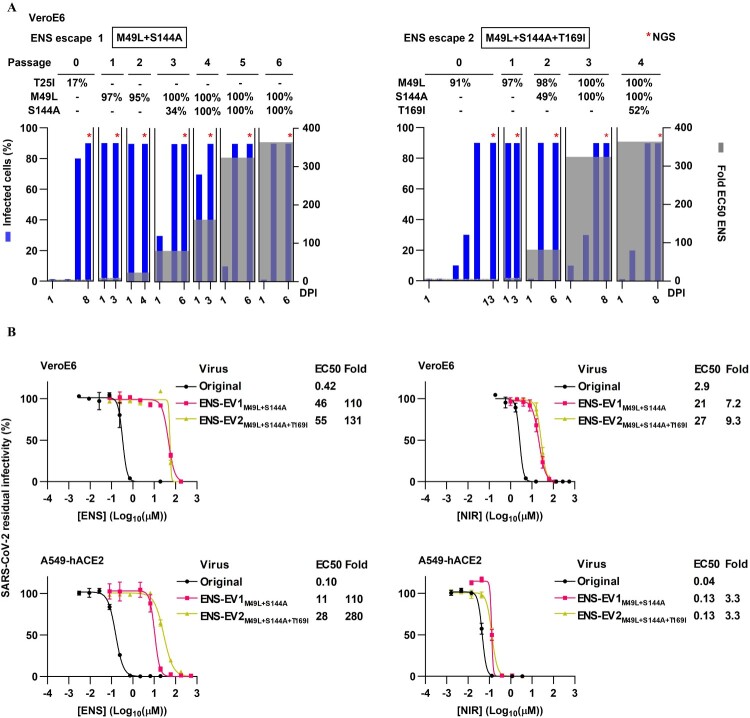
Selection of SARS-CoV-2 ensitrelvir resistant escape viruses with limited cross-resistance to nirmatrelvir. (A) SARS-CoV-2 escape from ensitrelvir (ENS) was induced in two serial passage experiments under increasing inhibitor concentrations. ENS escape 1 involved a primary escape culture (0) followed by six passages (1–6); ENS escape 2 involved a primary escape culture (0) and four passages (1–4). *, viral genomes in supernatants were analysed by next-generation sequencing (NGS). Identified Mpro substitutions and their frequencies (%) per passage are shown on top of the graphs. −, frequency <10%. Left y-axes, % of SARS-CoV-2-infected cells, estimated by immunostaining. Right y-axes, applied fold 50% effective concentrations (EC50) ensitrelvir. X-axes, day post infection (DPI). Further details in Supplementary Table S1. (B) Treatment of ensitrelvir escape viruses (ENS-EV1 and ENS-EV2) in VeroE6 or A549-hACE2 cells with ENS or nirmatrelvir (NIR). Residual infectivity was determined by relating counts of infected cells in treated cultures to means of counts of infected cells in non-treated cultures; values are means of four replicates with SEM. Concentration-response curves and EC50 were generated using GraphPad Prism 10.1.2. Fold resistance (Fold) was determined as EC50_variant Virus_/EC50_original Virus_ using rounded values; EC50_original Virus_ was a mean of EC50 from four to 12 independent experiments.

To identify ensitrelvir RAS, we serially passaged SARS-CoV-2 (isolate AHH1 [36]) in VeroE6 cells under increasing non-cytotoxic inhibitor concentrations (Figure 1(A), Supplementary Table S1 and Figure S1). Two such viral escape experiments were analysed to track evolution of putative RAS by NGS. M49L in Mpro emerged initially, while S144A co-emerged when treatment concentrations were increased to 81-fold EC50 of ensitrelvir and persisted until application of 364-fold EC50 of ensitrelvir, the highest applied concentration. At this concentration, and only in the second escape experiment, T169I was co-selected. Of note, in both escape experiments, viruses escaped 364-fold (153 μM) but not 445-fold EC50 (187 μM) ensitrelvir.

The polyclonal virus population from the first escape experiment (ENS-EV1 with M49L and S144A in Mpro; collected after one inhibitor free passage) showed up to 110-fold resistance against ensitrelvir in antiviral assays in VeroE6 monkey kidney or A549-hACE2 human lung cells, while the polyclonal virus population from the second escape experiment (ENS-EV2 with M49L, S144A and T169I in Mpro; collected after one inhibitor free passage) showed up to 280-fold resistance (Figure 1(B)). Interestingly, these polyclonal ensitrelvir escape viruses showed limited (<10-fold) cross-resistance to nirmatrelvir.

### Highly fit SARS-CoV-2 recombinants with high resistance to ensitrelvir but limited cross-resistance to nirmatrelvir

**Figure 2.**
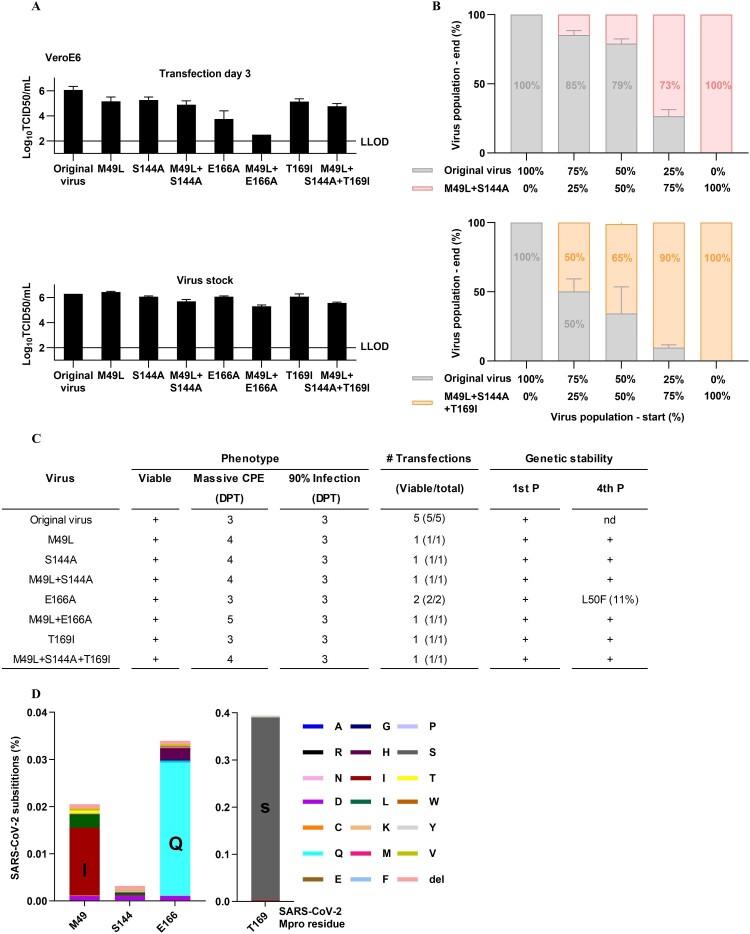
High fitness of recombinant SARS-CoV-2 variants with ensitrelvir RAS and natural occurrence of these RAS. (A) Infectivity titres of recombinant variants with specified RAS, determined in supernatants from day 3 post transfection or virus stocks in VeroE6 cells. 50% tissue culture infectious doses (TCID50)/mL are means of four replicates with SEM. LLOD, lower limit of detection. (B) Competition growth assays following infection with the original virus and the M49L + S144A variant (top) or the original virus and the M49L + S144A + T169I variant (bottom). X axes, ratios of the specified virus populations at the start of the experiment. Y axes, ratios of virus populations at the end of the experiment, following viral spread to the majority of culture cells, as determined by NGS; virus populations are colour-coded. (C) Other characteristics of recombinant variants: (i) Phenotype: viability and day post transfection (DPT), at which massive cytopathogenic effects (CPE) and 90% infection, determined by immunostaining, were observed; (ii) # Transfections: number of viable versus total transfections; (iii) Genetic stability: Mpro stability in first (1st) and fourth (4^th^) viral passage (P), +, no non-synonymous mutations in Mpro in >10% of genomes, nd, not done. Further details in Supplementary Table S2 and S3. (D) Percentage of viruses that differed genetically from the consensus of 17,007,971 Mpro sequences retrieved from the GISAID database on October 11, 2024, at the specified position. Specific amino acid residues have distinct colours; del, deletion. Further details in Supplementary Table S4 and S5.

We next carried out reverse genetic studies [32] for the identified putative ensitrelvir RAS. Cell culture infectious SARS-CoV-2 recombinants with M49L, S144A and T169I, singly or in the observed combinations, showed high viral fitness, as evidenced by infectivity titres in transfection and viral passage cultures that were comparable to those of the original polyclonal virus (Figure 2(A)). In competition growth assays, the original virus showed slightly higher fitness than the M49L + S144A variant, while the M49L + S144A + T169I variant showed somewhat higher fitness than the original virus (Figure 2(B)). Importantly, none of the resistant variants were outcompeted by the original virus in these co-cultures. For a head-to-head comparison, we also studied E166A singly and in combination with M49L, both recently linked to ensitrelvir resistance [52]. E166A and E166A + M49L conferred a higher fitness cost than the RAS identified in our study (Figure 2(A)). In all recombinant variants generated, Mpro with RAS was genetically stable following four viral passages, except for the E166A variant, which acquired L50F in 11% of viral genomes (Figure 2(C), Supplementary Table S2 and S3). This high fitness is in line with the observation of some genetic flexibility at Mpro-positions 49, 144, 166 and 169 and natural occurrence of these RAS, especially M49L and T169I, which were present in >400 sequences deposited in the GISAID database in October 2024 (Figure 2(D) and Supplementary Table S4).

**Figure 3.**
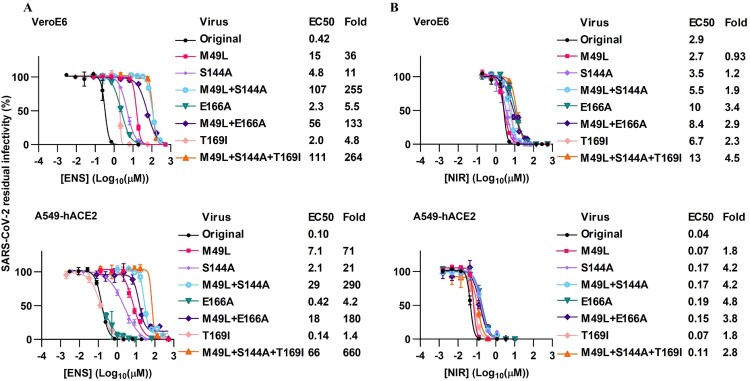
SARS-CoV-2 variants with ensitrelvir RAS were highly resistant to ensitrelvir, but not to nirmatrelvir. Treatment of recombinant SARS-CoV-2 variants with specified ensitrelvir RAS in VeroE6 or A549-hACE2 cells with (A) ensitrelvir (ENS) or (B) nirmatrelvir (NIR). For details on determination of residual infectivity values, concentration-response curves, EC50 and fold resistance see Figure 1(B) legend.

In antiviral assays, among single substitutions, M49L conferred the highest ensitrelvir resistance (up to 71-fold), followed by S144A (up to 21-fold) and T169I (up to 4.8-fold) (Figure 3(A)). These substitutions interacted in a synergistic way. Thus, the combination of M49L and S144A conferred up to 290-fold resistance. Addition of T169I to this combination resulted in a triple mutant with up to 660-fold resistance. M49L + E166A only conferred up to 180-fold resistance, with the two substitutions showing synergy.

All investigated ensitrelvir RAS conferred <5-fold cross-resistance to nirmatrelvir in the cell lines tested (Figure 3(B)).

### Nirmatrelvir resistant SARS-CoV-2 variants showed cross-resistance to ensitrelvir

**Figure 4.**
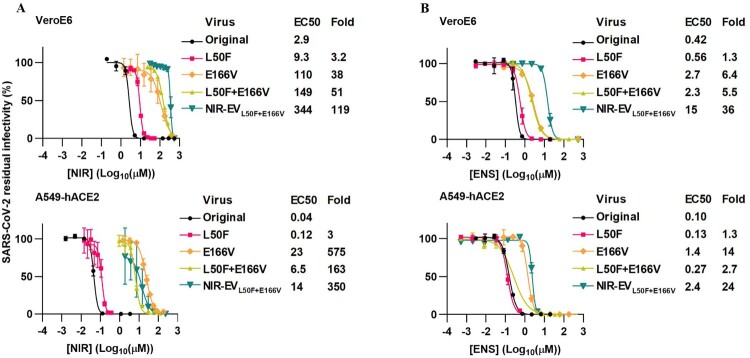
Nirmatrelvir resistant SARS-CoV-2 variants were resistant to ensitrelvir. Treatment of recombinant variants with specified nirmatrelvir RAS and of the polyclonal nirmatrelvir escape virus NIR EV_L50F_ _+_ _E166V_ [22] in VeroE6 or A549-hACE2 cells with (A) nirmatrelvir (NIR) and (B) ensitrelvir (ENS). NIR data in (A) are reproduced from [22] to facilitate a better comparison. For details on determination of residual infectivity values, concentration-response curves, EC50 and fold resistance see Figure 1(B) legend.

The previously reported polyclonal nirmatrelvir escape virus, harbouring the nirmatrelvir RAS E166V in combination with the fitness compensating substitution L50F (Figure 4(A) [22]), showed up to 36-fold cross-resistance to ensitrelvir (Figure 4(B)). Recombinant variants with E166V, singly or in combination with L50F (Figure 4(A) [22]), showed up to 14-fold ensitrelvir cross-resistance (Figure 4(B)). These observations suggested an asymmetrical cross-resistance pattern for ensitrelvir and nirmatrelvir, where nirmatrelvir RAS showed greater resistance to ensitrelvir compared to the converse.

### Differential impact of ensitrelvir RAS on SARS-CoV-2 replication and Mpro function

**Figure 5.**
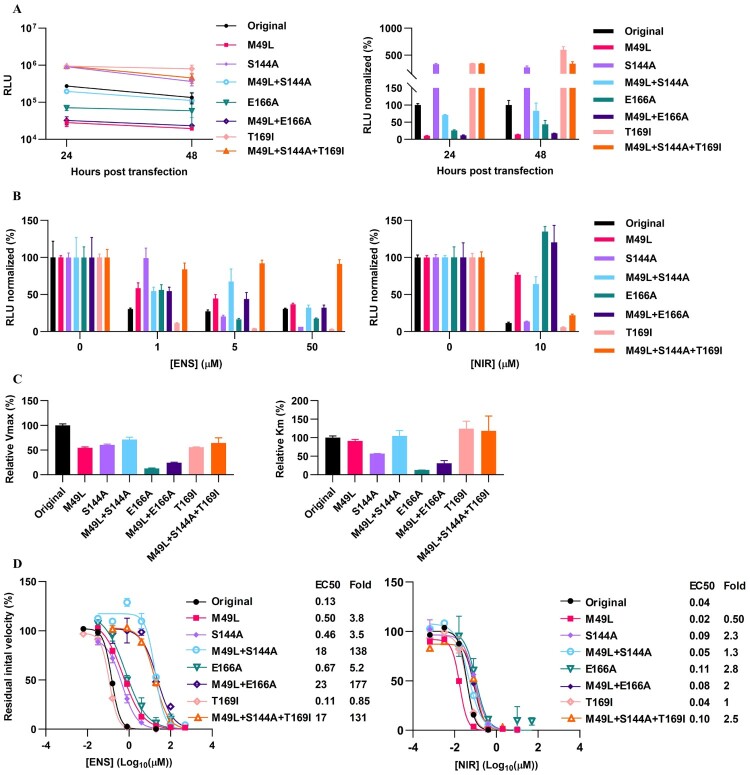
Effect of ensitrelvir RAS on SARS-CoV-2 replication and Mpro activity. (A) Luciferase activity of replicons with specified ensitrelvir RAS in VeroE6 cells. Left, relative light units (RLU) 24- and 48-h post transfection. Right, % RLU relative to the mean RLU of the original replicon. (B) % RLU in cultures treated with ensitrelvir (ENS) (left) or nirmatrelvir (NIR) (right), relative to mean RLU of the respective non-treated cultures. (A and B) Values are means of six replicates with SEM. (C) Enzyme kinetics of SARS-CoV-2 Mpro variants in a FRET assay. Relative Vmax (left) and relative Km (right) were determined by relating Vmax and Km of the Mpro variants to the Vmax and Km of the original Mpro determined in the same experiment. Km and Vmax were determined in Graphpad Prism 10.1.2. Further details in Supplementary Figure S2. (D) Residual initial velocity of treated Mpro variants, relative to the mean initial velocity of the non-treated respective Mpro. For details on determination of concentration-response curves, EC50 and fold resistance see Figure 1(B) legend. (C and D) Values are means of two replicates with SEM.

We further investigated the effect of ensitrelvir RAS in the context of SARS-CoV-2 replication [32]. In line with assays with cell culture infectious recombinants, replicons harbouring E166A or M49L + E166A showed low replication fitness (Figure 5(A)). However, M49L apparently had a stronger negative impact in the replicon than in the infectious virus assay, while S144A, T169I and M49L + S144A + T169I increased replication capacity (Figure 5(A)).

Additionally, in line with results in cell culture infectious recombinants, replicons with M49L + S144A + T169I showed the highest resistance to ensitrelvir, while replicons with M49L + E166A showed lower resistance (Figure 5(B)). However, E166A and M49L + E166A conferred relatively high resistance to nirmatrelvir.

The effects of ensitrelvir RAS on Mpro enzymatic function [22] were in line with those in cell culture infectious assays and partly in line with those in replicon assays (Figure 5(C,D)). While E166A and M49L + E166A had the strongest negative impact on Mpro function, most of the other substitutions had a limited negative impact (Figure 5(C) and Supplementary Figure S2). In Mpro enzymatic assays, only the combinations of RAS (M49L + S144A, M49L + S144A + T169I and M49L + E166A) conferred strong resistance to ensitrelvir, while none of the tested RAS conferred resistance to nirmatrelvir; there was a positive correlation between EC50 values determined in Mpro enzymatic and cell-based antiviral assays (Figure 5(D) and Supplementary Figure S3).

### Molecular dynamics simulations revealed key interactions in SARS-CoV-2 Mpro-ensitrelvir complexes

**Figure 6.**
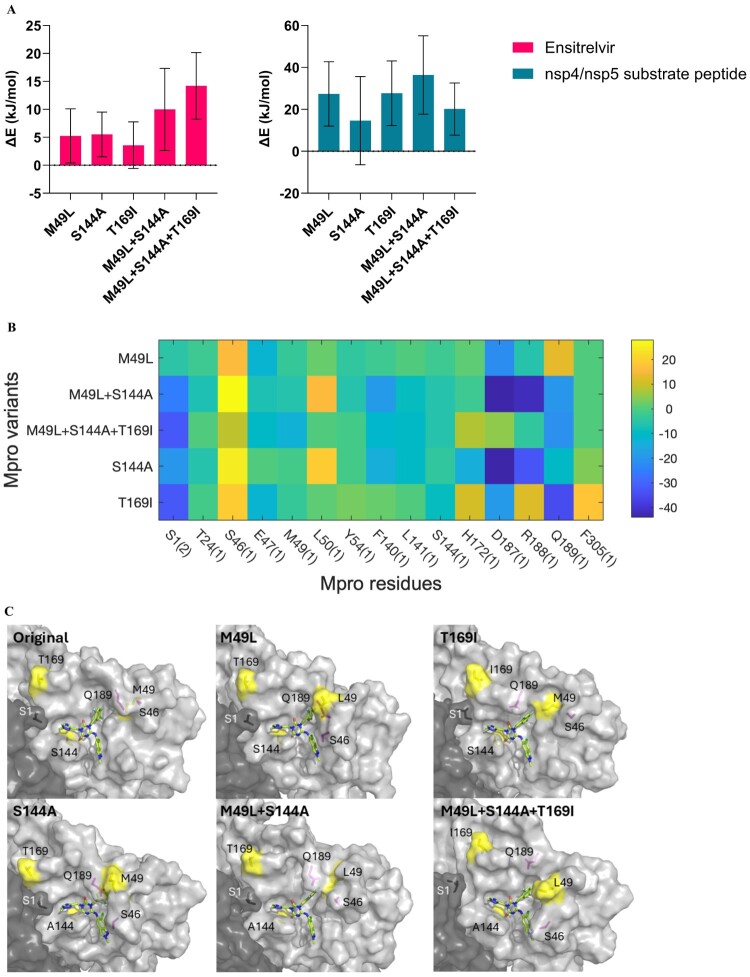
Key interactions in SARS-CoV-2 Mpro complexes revealed through molecular dynamics simulations. (A) Interaction energy differences (ΔE = E_variant Mpro_ – E_original Mpro_) for binding of ensitrelvir (ENS, left) or nsp4/nsp5 substrate peptide (right) to Mpro variants with M49L, S144A, T169I, M49L + S144A or M49L + S144A + T169I substitutions, as determined from triplicate MDS. (B) Contact heatmap visualizing changes in contact frequencies between ensitrelvir and Mpro caused by substitutions in Mpro (M49L, M49L + S144A, M49L + S144A + T169I, S144A or T169I). Residues within 3 Å of ensitrelvir were defined as being in contact with the inhibitor, and they are represented on the x-axis with (1) used for residues in the monomer binding to ensitrelvir and (2) used for residue S1 in the neighbour monomer. The contact frequencies are relative to the original Mpro (Δ*f* = *f*_variant Mpro_ – *f*_original Mpro_). Positive values (yellow) indicate a gain in contacts compared to original Mpro, while negative values (dark blue) represent a loss of contacts. (C) Representative snapshots to visualize the substrate pocket in ensitrelvir-Mpro complexes extracted from the final frames of the MDS. Residues 49, 144, and 169 are presented in yellow, key interactions with residues 46 and 189 in magenta, and ensitrelvir in green. Monomer (1) and monomer (2) are represented in light grey and dark grey, respectively.

MDS were carried out to study the Mpro-ensitrelvir and the Mpro-substrate peptide complexes at an atomic level. The stability of the simulations was confirmed by monitoring structural properties including the root-mean-squared deviations of the backbone atoms (Supplementary Figures S4–S11). For the Mpro-ensitrelvir complexes, the interaction energy differences were largest for Mpro with RAS combinations M49L + S144A or M49L + S144A + T169I, suggesting that these substitutions resulted in a lower affinity to and less favourable interactions with ensitrelvir (Figure 6(A)). This correlates well with the findings from the in vitro assays, where the M49L + S144A and M49L + S144A + T169I variants showed the highest resistance to ensitrelvir.

The weakened interactions between ensitrelvir and Mpro with the M49L + S144A or M49L + S144A + T169I substitutions could be explained by changes in the structural conformation of the substrate pocket, as consistently observed across all three independent MDS, each lasting 200 ns. A key interaction in the Mpro dimer is the stabilization of the *S1* substrate pocket by the N-terminus of the neighbouring monomer, S1, through a hydrogen-bonding network. As a non-covalent inhibitor, ensitrelvir relies on the stability of the hydrogen-bond network to interact with Mpro [53,54]. The MDS revealed that, in Mpro with the M49L + S144A or M49L + S144A + T169I substitutions, S1 retracted from the substrate pocket, thereby destabilizing the pocket, which partly explained the weakened ensitrelvir binding (Figure 6(B) and Figure 6(C)). In addition, the structural rearrangement of the substrate pocket of Mpro harbouring the M49L + S144A or M49L + S144A + T169I substitutions was characterized by an increase in ensitrelvir interactions with S46 and a decrease in interactions with Q189, thus deviating from the native binding conformation in the original Mpro.

The MDS suggested that the ensitrelvir RAS led to decreased or unchanged substrate binding (Figure 6(A)).

### Substitutions at resistance hotspot Mpro-position 166 had differential impact on SARS-CoV-2 fitness and resistance

**Figure 7.**
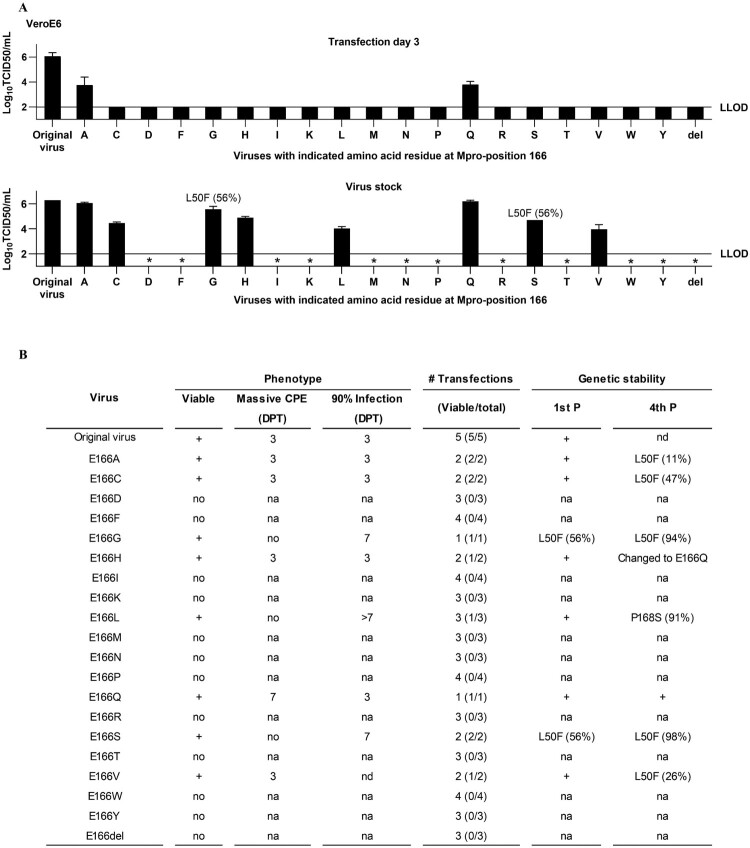
Differential fitness cost of substitutions at resistance hotspot SARS-CoV-2 Mpro-position 166. (A) Infectivity titres of recombinant position 166 variants in supernatants from day 3 post transfection or virus stocks in VeroE6 cells. 50% tissue culture infectious doses (TCID50)/mL are means of four replicates with SEM. del, deletion. LLOD, lower limit of detection. *, not done. Titres of E166V are reproduced from [22]. (B) Other characteristics of position 166 variants: (i) Phenotype: viability and day post transfection (DPT), at which massive cytopathogenic effects (CPE) and 90% infection, determined by immunostaining, were observed; (ii) # Transfections: number of viable versus total transfections; (iii) Genetic stability: Mpro stability in first (1st) and fourth (4th) viral passage (P), +, no non-synonymous mutations in Mpro in >10% of genomes, na, not applicable, nd, not done. Further details in Supplementary Table S6 and S7.

**Figure 8.**
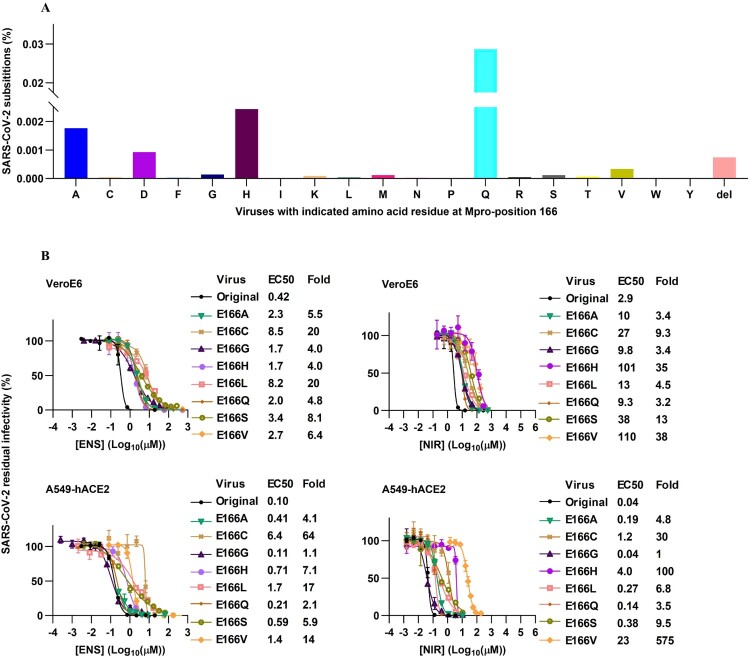
Natural genetic variation at SARS-CoV-2 Mpro-position 166 and differential effects of substitutions on viral sensitivity to Mpro inhibitors. (A) Percentage of viruses that differed genetically from the consensus of 17,007,971 Mpro sequences retrieved from the GISAID database on October 11, 2024, at Mpro-position 166. Further details in Supplementary Table S4 and S5. (B) Treatment of recombinant variants with specified Mpro-position-166-substitutions in VeroE6 or A549-hACE2 cells with ensitrelvir (ENS) or nirmatrelvir (NIR). The data for the E166V variant treated with nirmatrelvir are reproduced from [22] to ease comparison. For details on determination of residual infectivity values, concentration-response curves, EC50 and fold resistance see Figure 1(B) legend.

As Mpro-position 166 had been linked to both ensitrelvir and nirmatrelvir resistance [22–26,52], we investigated the impact of different substitutions at this position on viral fitness and Mpro inhibitor resistance. We engineered recombinants with all possible substitutions and a deletion of Mpro-position 166. Among these recombinants, only the ones harbouring E166A/C/G/H/L/Q/S/V were viable, but each incurred a specific fitness cost compared to the original virus (Figure 7(A,B)). This included the two substitutions most frequently occurring in patient isolates, E166H/Q (Figure 8(A) and Supplementary Table S4 and S5). Interestingly, viable recombinants with E166A/C/G/S/V had acquired L50F following first or fourth viral passage, while E166L had acquired P168S following the fourth passage. E166H had changed to E166Q following the fourth passage (Figure 7(B), Supplementary Table S6 and S7).

Resistance testing of the viable recombinants, using first passage inhibitor free virus stocks, revealed a differential impact of specific substitutions on ensitrelvir versus nirmatrelvir sensitivity (Figure 8(B)). High (≥20-fold resistance in at least one cell line) was conferred by E166C/L to ensitrelvir and by E166C/H/V to nirmatrelvir. Specifically, E166C conferred up to 64-fold and E166L up to 20-fold ensitrelvir resistance, while E166C conferred up to 30-fold, E166H up to 100-fold and E166V up to 575-fold nirmatrelvir resistance. Including these observations, > 5-fold resistance to both drugs was conferred by E166C/H/L/S/V in at least one cell line. E166G/Q conferred <5-fold resistance to any of the two drugs in any of the two tested cell lines. The cumulative resistance conferred by any substitution at position 166 in both cell lines, was lower for ensitrelvir (188-fold) than nirmatrelvir (840-fold), largely due to high resistance conferred by E166H/V to nirmatrelvir.

## Discussion

In this study, we identified highly fit and resistant SARS-CoV-2 ensitrelvir escape variants. These variants showed limited cross-resistance to nirmatrelvir, while nirmatrelvir-resistant variants showed significant cross-resistance to ensitrelvir. We uncovered the functional landscape of amino acid variation at Mpro-position 166, including naturally occurring polymorphisms.

Resistance and fitness levels of the newly identified M49L+S144A and M49L+S144A+T169I ensitrelvir-resistant variants exceeded those of a variant harbouring RAS M49L+E166A. These latter RAS were recently selected in cell culture infectious SARS-CoV-2 under ensitrelvir pressure and were included in our study to facilitate a head-to-head-comparison [52]. Our detailed evolutionary analysis of RAS emergence under selective pressure, coupled with the selection of M49L in two independent selection studies [52, 55] using cell culture infectious viruses, underscores the pivotal role of this substitution for ensitrelvir resistance. Nevertheless, high resistance apparently depended on a combination of this RAS with synergistic RAS. In our study, highly resistant variants harbouring synergistic RAS were selected when at least 34 mM (81-fold EC50) ensitrelvir was applied, which exceeds concentrations applied in two independent studies of up to 5 mM and 1.1 mM, respectively [52, 55]. Importantly, concentrations applied in our study are in line with physiologically relevant concentrations in COVID-19 patients treated with a standard dose regimen (day 1: 375 mg; day 2-5: 125 mg), where mean plasma concentrations of around 37 mM and a maximum serum concentration (Cmax) of 57 mM were recorded in Japanese patients [56]. The RAS selected in our study likely confer clinically relevant resistance, as the ensitrelvir Cmax/EC50 ratio for the original virus (136 and 570 in VeroE6 and A549-hACE2 cells, respectively) was greatly reduced for the polyclonal ensitrelvir escape viruses and the variants with M49L+S144A or M49L+S144A+T169I (to ≤1.2 in VeroE6 and ≤5.2 in A549-hACE2 cells, respectively).

Remarkably, we report an asymmetrical cross-resistance pattern for ensitrelvir and nirmatrelvir RAS. Specifically, nirmatrelvir RAS exhibited a higher degree of resistance to ensitrelvir compared to the reverse. Ensitrelvir cross-resistance conferred by nirmatrelvir RAS is likely clinically relevant, as the ensitrelvir Cmax/EC50 ratio of 136 and 570 for the original virus was reduced to 4 and 24 for the polyclonal nirmatrelvir escape virus in VeroE6 and A549-hACE2 cells, respectively.

Ensitrelvir-resistant variants showed high fitness and co-circulated together with the original virus in competition growth assays. In contrast, the nirmatrelvir-resistant variants were outcompeted by the original virus in the same assays [22]. The low fitness cost of the newly identified ensitrelvir RAS could translate into their persistence following treatment and spread of the respective variants in human populations. Indeed, the frequency of the ensitrelvir RAS M49L appears to have increased since its introduction to the market in Japan, where >1 million people have been treated. Thus, prior versus after market introduction (May versus October 2023), 60% versus 88% of all recorded M49L originated from Japan [55]; in October 2024, 77% of M49L originated from Japan (own GISAID analysis). For the nirmatrelvir RAS E166V a 6.9-fold increase in the percentage has been recorded globally between April 2022 and October 2024, while absolute prevalence remained low (Supplementary Table S5). A similar increase in the percentage of E166V was also reported by a recent study [57]. The barrier to resistance appears to be higher for nirmatrelvir than for ensitrelvir, as the in vitro acquisition of E166V was more time-consuming than that of M49L [22], and as the frequency of E166V in patients is lower than that of M49L [57,58].

Based on findings by us and others, Mpro-position 166 has emerged as a hotspot for resistance to the structurally different ensitrelvir and nirmatrelvir, underscoring an important role of this position in drug-target interactions [22–26,52]. We found that specific substitutions at position 166 had differential impact on viral fitness and resistance to ensitrelvir versus nirmatrelvir. Importantly, the most frequently naturally occurring substitution E166Q did not confer significant resistance to any of these two inhibitors, while the second most frequent naturally occurring substitution E166H conferred high resistance to nirmatrelvir. These findings are in line with a previous report on the effect of Mpro polymorphisms on nirmatrelvir resistance [59]. One recent structure-based study suggested that the nirmatrelvir resistance conferred by changes at E166 is caused by the loss of the hydrogen bond between E166 and the pyrrolidone ring [60]. While the frequency of pre-existing RAS remains low, they could accelerate the spread of resistance in populations given a widespread inhibitor use. Overall, the efficacy of nirmatrelvir was more affected by different changes at position 166 than that of ensitrelvir. The critical role of position 166 for viral fitness was underscored by the reduced fitness of all investigated position 166 variants. Non-viability of certain position 166 variants, such as variants with E166D and E166del, despite natural occurrence of the respective substitutions in patient isolates, could be explained by sequencing artefacts or by the co-selection of fitness-compensating substitutions in these isolates. Interestingly, findings in our study suggest L50F, previously characterized as a fitness-compensating substitution for E166V and A173V in nirmatrelvir resistant variants and co-selected with E166V in nirmatrelvir treated patients [22,23,31,42], to be a versatile fitness compensator, capable of mitigating fitness costs associated with different substitutions at Mpro-position 166. This aligns with a recent study using full-length Mpro substrates to test the effect of L50F in various Mpro variants [29]. The study demonstrated that L50F could compensate for the loss of fitness caused by other RAS in the active site of the SARS-CoV-2 Mpro [29]. L50F was shown to enhance the dimer-dimer interactions, thereby stabilizing the dimer structure of Mpro and ensuring that the enzyme maintained its functional conformation [29].

Nirmatrelvir RAS (L50F and E166V) identified in the in vitro systems used in our study were detected in patients who failed treatment validating their clinical relevance [25,26,61]. The ensitrelvir RAS M49L and S144A identified in this study were also detected in treated patients; however, it remains to be investigated if these RAS are associated with treatment failure [58]. Studies in the Syrian golden hamster model could provide insights into their impact on in vivo fitness, pathogenicity and resistance [52,62].

This comparative analysis of resistance and cross-resistance profiles of the two clinically most relevant oral SARS-CoV-2 Mpro inhibitors offers valuable insights for COVID-19 treatment strategies. Given the conserved nature of the coronavirus Mpro, these findings further contribute to pandemic preparedness and can guide the design of next-generation protease inhibitors.

## Author contributions

YZ, JMG contributed to the conception and design of the study. YZ, KAG, HDT, LAR, AO, AC, ZD, LVP, UF, GHJP, SR, JB, JMG generated assays and experimental data. Data was verified by GHJP, SR, JB, JMG. YZ, KAG, HDT, LAR, JMG drafted the original manuscript. All authors read and approved the final version of the manuscript.

## Supplementary Material

Supplemental Material

## Data Availability

All data are available in the main text or the supplementary material.
