# Supplementary material for: SARS-CoV-2 Mpro inhibitor ensitrelvir: asymmetrical cross-resistance with nirmatrelvir and emerging resistance hotspots
Source: Emerg Microbes Infect. 2025 Oct 14;14(1):2552716. doi: 10.1080/22221751.2025.2552716 (PMC12529750; doi:10.1080/22221751.2025.2552716)
Supplement: Supplemental Material [file TEMI_A_2552716_SM7430.docx]

**Supplemental material**

**Supplementary Figures**


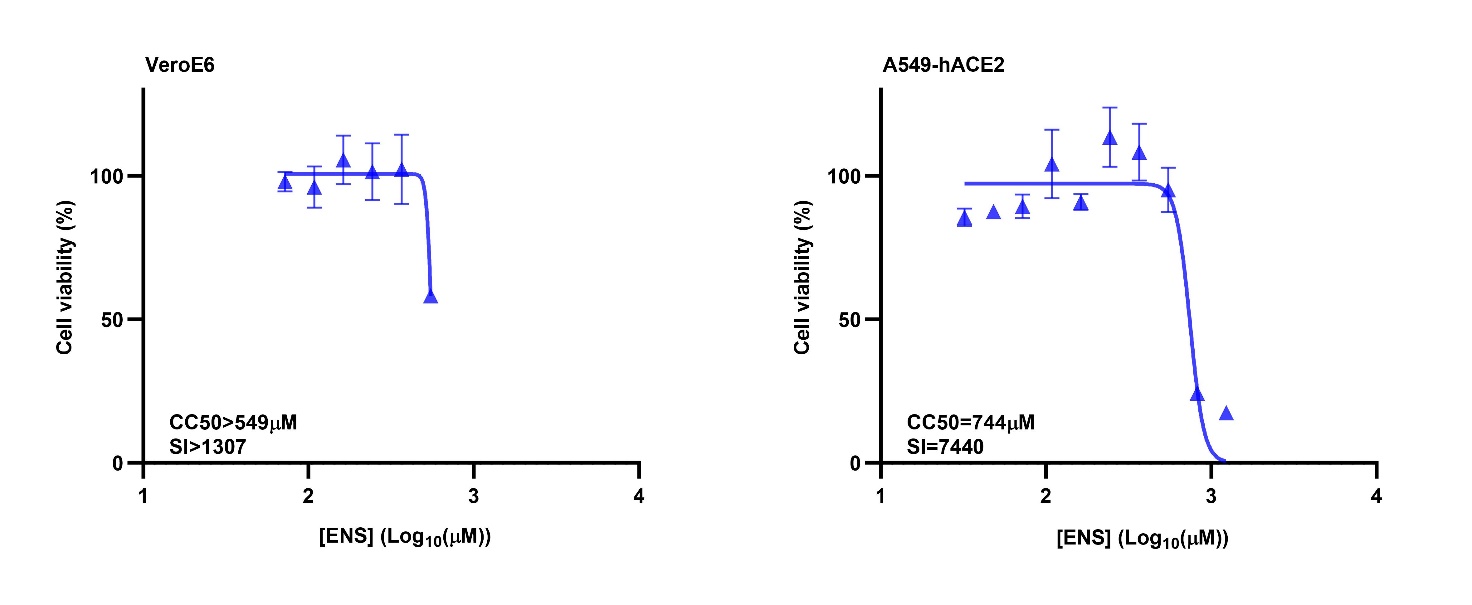
 **Supplementary Figure S1. Ensitrelvir exhibited low cytotoxicity and a high selectivity index.** Cell viability assays for ensitrelvir (ENS) were carried out in VeroE6 and A549-hACE2 cells using 96-well plates. The % cell viability was calculated by relating the OD values of treated cultures to the mean OD of 10 non-treated control cultures and are presented as means of 3 replicates. Curves and 50% cytotoxic concentrations (CC50) were generated using GraphPad Prism 10.1.2. The selectivity index (SI) was calculated as the ratio of CC50 to 50% effective concentration (EC50).


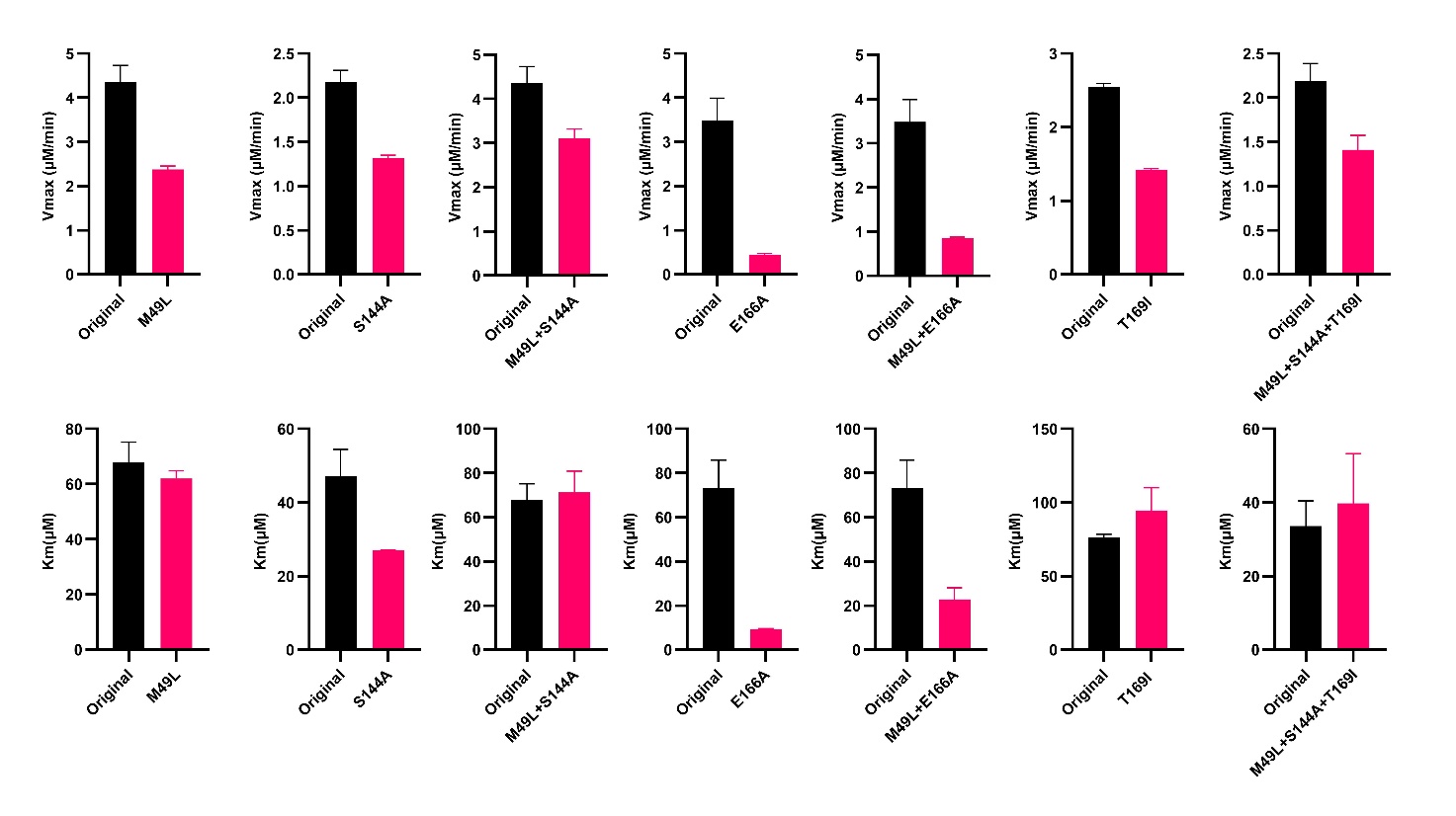


**Supplementary Figure S2. Vmax and Km values of SARS-CoV-2 Mpro variants.**

For the specified Mpro variants, the maximum velocity (Vmax) and the Michaelis Menten constant (Km) were determined using the Michaelis Menten equation V_0_=(V_max_×[S_0_])/(K_m_+[S_0_]), in GraphPad Prism 10.1.2 and are given as means of two replicates with SEM. In each experiment, the original Mpro was included together with the specified Mpro variant, enabling determination of relative Vmax and Km values included in Figure 5(C).

**
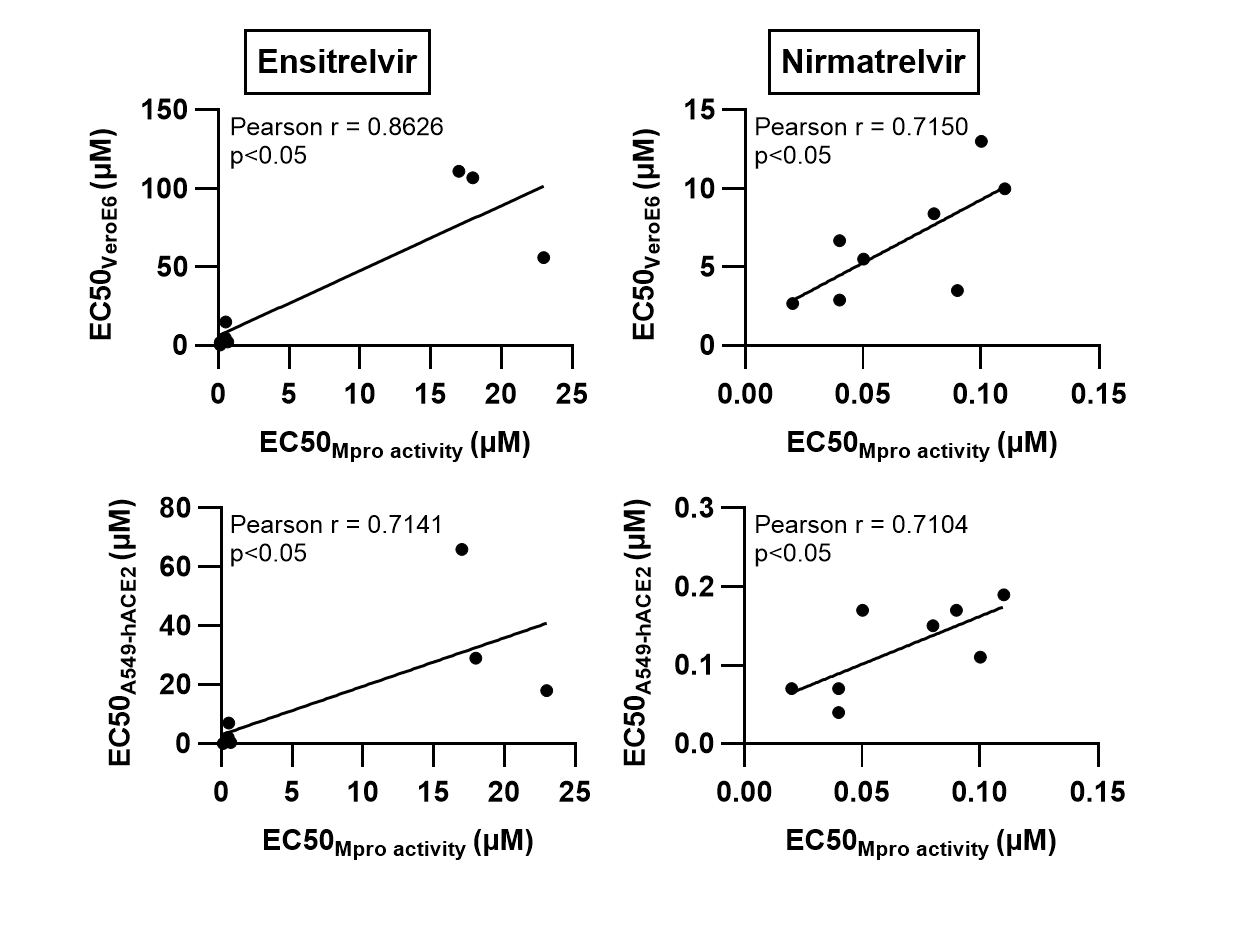
**

**Supplementary Figure S3. Correlation between EC50 values determined in Mpro enzymatic assays and EC50 values determined in cell-based antiviral assays.**

Shown are correlations between EC50 of ensitrelvir or nirmatrelvir determined in FRET -based Mpro enzymatic assays and in cell-based antiviral assays carried out in VeroE6 cells or A549-hACE2 cells as specified in the y-axes titles. Pearson correlation constant (r) and p values were determined in GraphPad Prism 10.1.2.


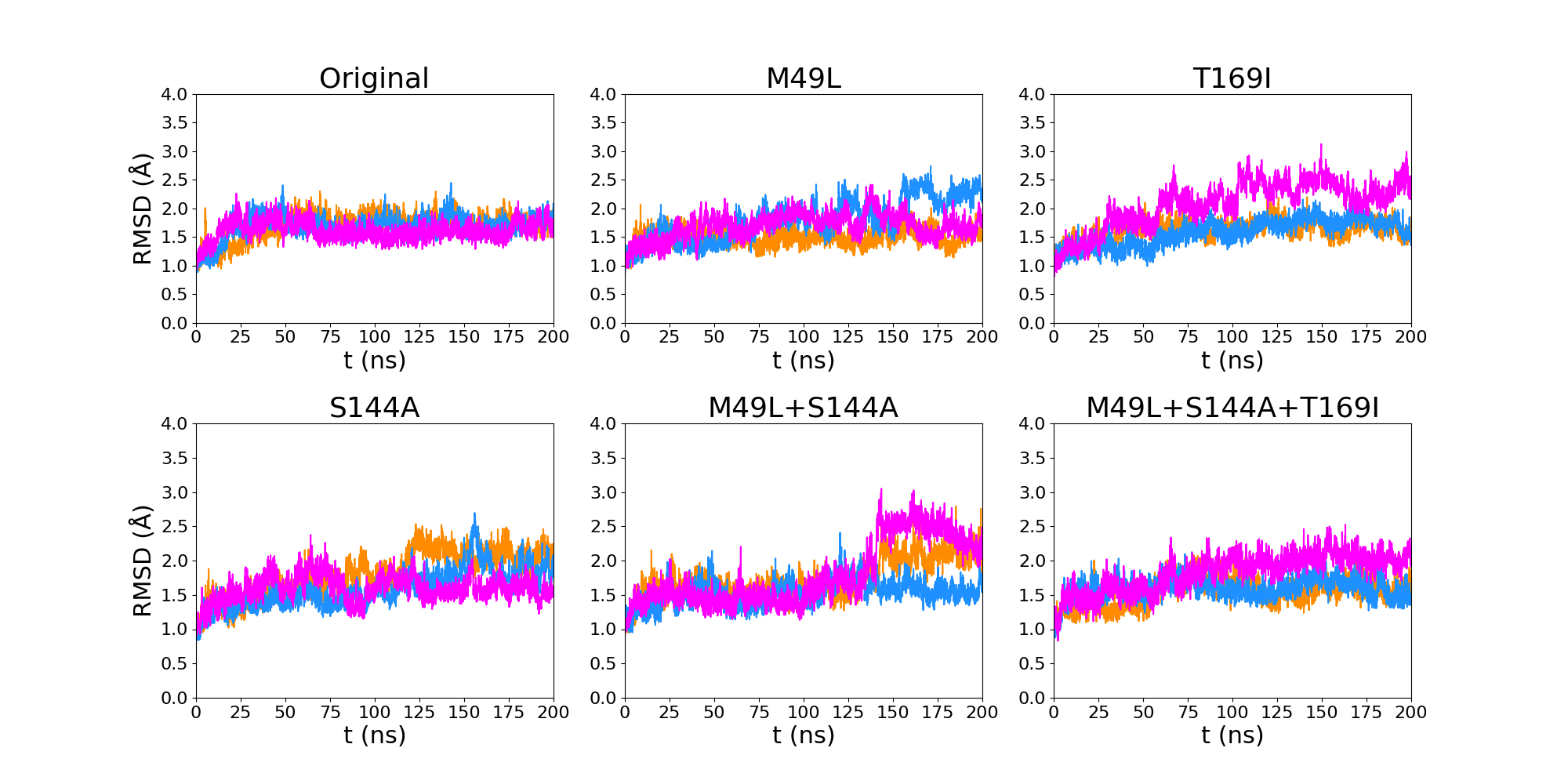


**Supplementary Figure S4. Time evolution of the root-mean-squared deviations (RMSD) for Mpro-ensitrelvir complexes during molecular dynamics simulations.**

RMSD were calculated with respect to the heavy backbone atoms in the energy-minimized structure specific to each of the three respective molecular dynamics simulations (MDS). Top row, left to right: Original Mpro; M49L; T169I. Bottom row, left to right: S144A; M49L+S144A; M49L+S144A+T169I. The triplicate simulations are represented in different colors. After approximately 50 ns, the RMSD values plateaued at around 2-3 Å for all complexes, indicating that the complexes were stable and that the substitutions did not induce global changes in protein conformation.


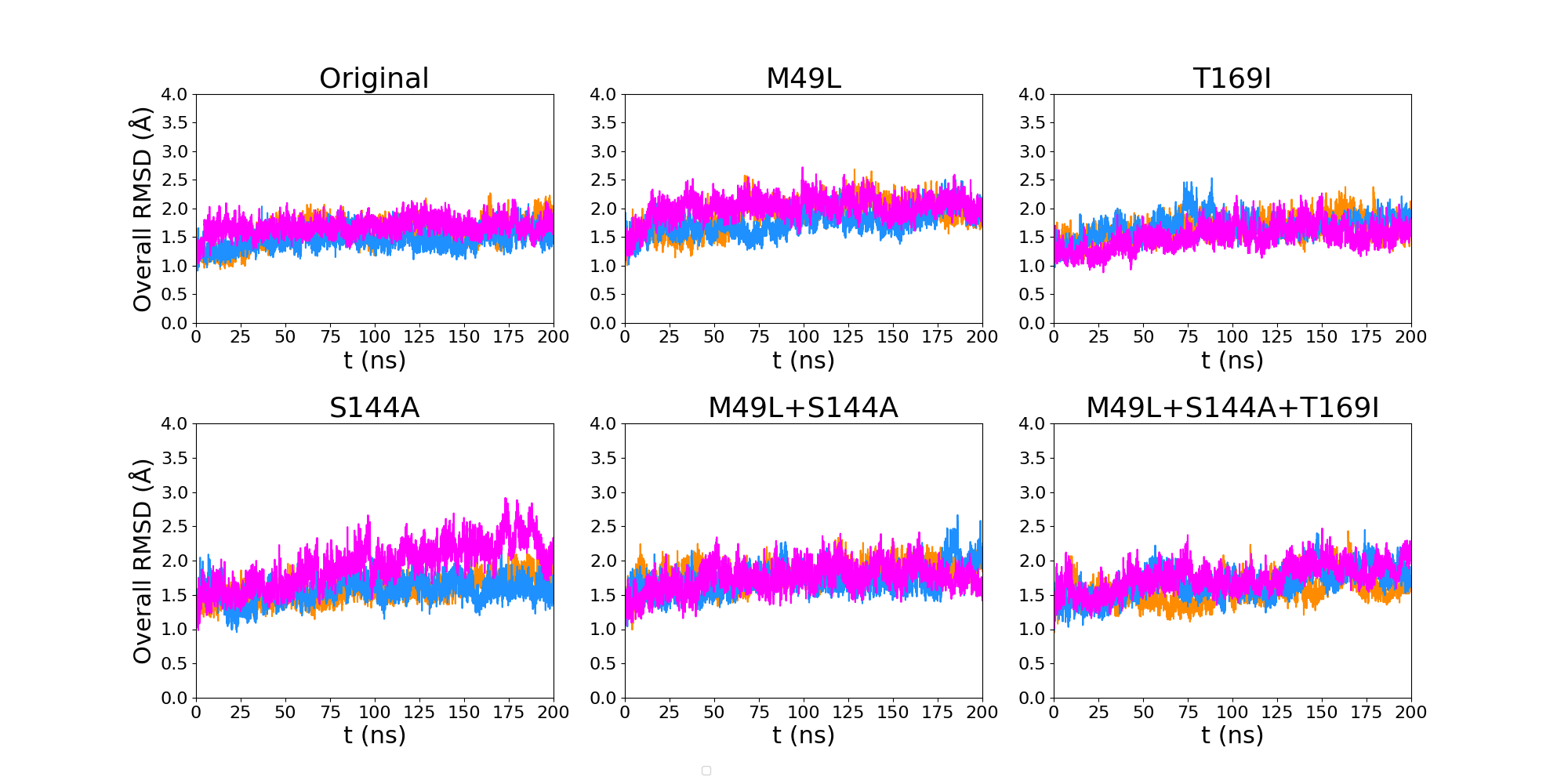


**Supplementary Figure S5. Time evolution of RMSD for Mpro-substrate peptide complexes during molecular dynamics simulations.**

RMSD were calculated with respect to the heavy backbone atoms in the energy-minimized structure specific to each of the three respective MDS. Top row, left to right: Original Mpro; M49L; T169I. Bottom row, left to right: S144A; M49L+S144A; M49L+S144A+T169I. The triplicate simulations are represented in different colors. After approximately 50 ns, the RMSD values plateaued at around 2-3 Å for all complexes, indicating that the complexes were stable and that the substitutions did not induce global changes in protein conformation.


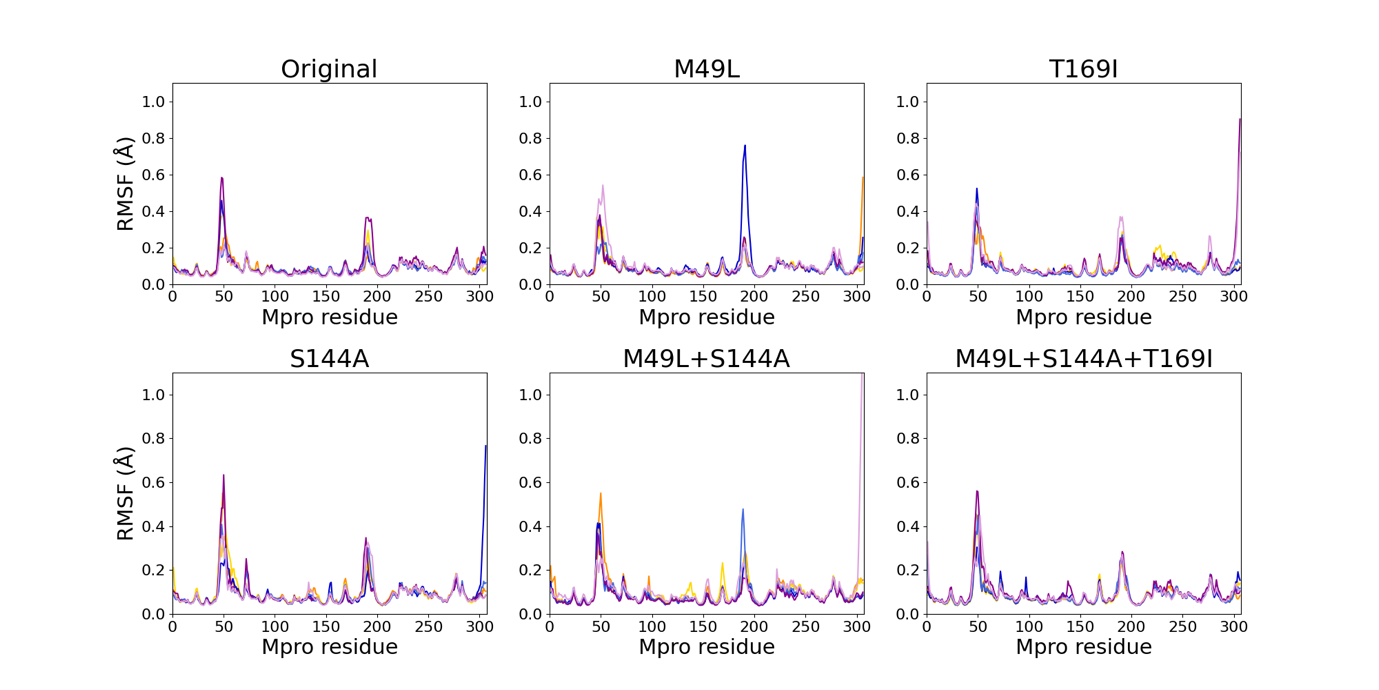


**Supplementary Figure S6. Averaged root-mean-squared fluctuations (RMSFs) for Mpro residues in Mpro-ensitrelvir complexes** **extracted from molecular dynamics simulations.**

RMSFs of the heavy backbone atoms were averaged across the three respective molecular dynamics simulations of each system. Top row, left to right: Original Mpro; M49L; T169I. Bottom row, left to right: S144A; M49L+S144A; M49L+S144A+T169I. Each monomer in each of the three MDS of dimers is represented in a different color.


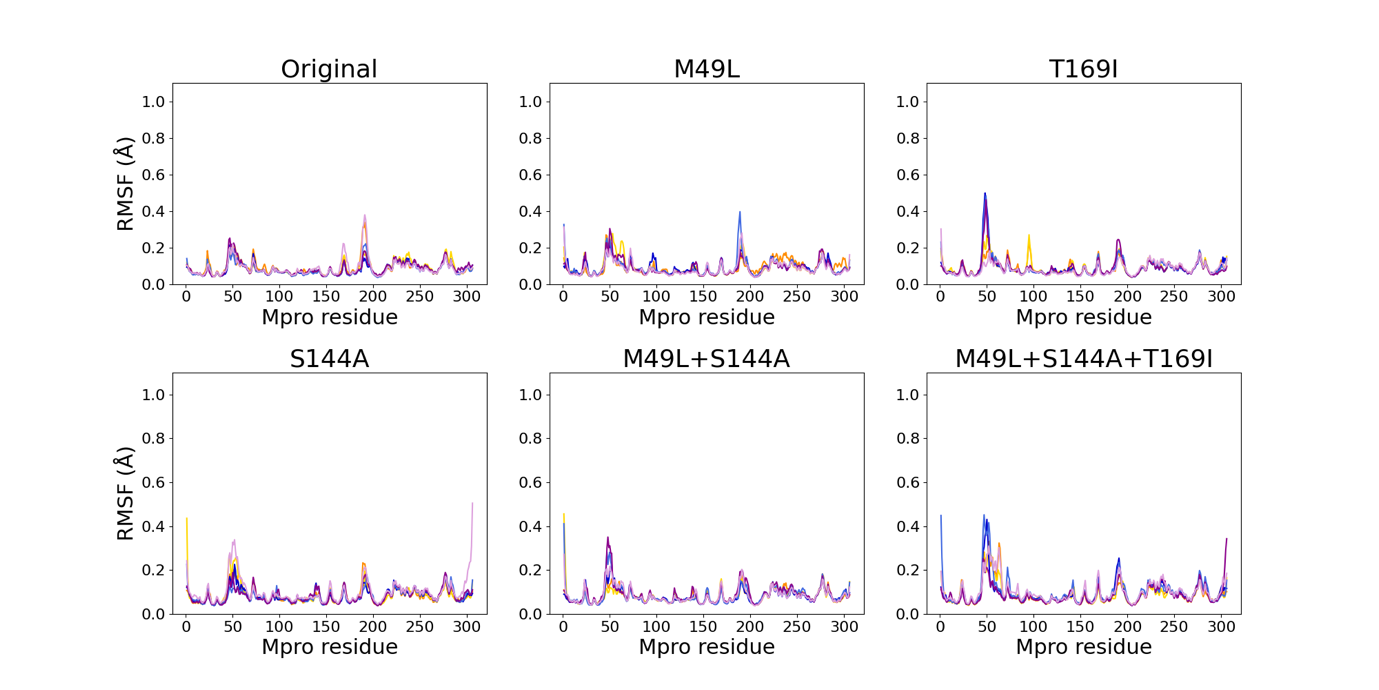
**Supplementary Figure S7. Averaged root-mean-squared fluctuations (RMSFs) for Mpro residues in Mpro-substrate peptide complexes extracted from molecular dynamics simulations.**

RMSFs of the heavy backbone atoms were averaged across the three respective molecular dynamics simulations of each system. Top row, left to right: Original Mpro; M49L; T169I. Bottom row, left to right: S144A; M49L+S144A; M49L+S144A+T169I. Each monomer in each of the three MDS of dimers is represented in a different color.


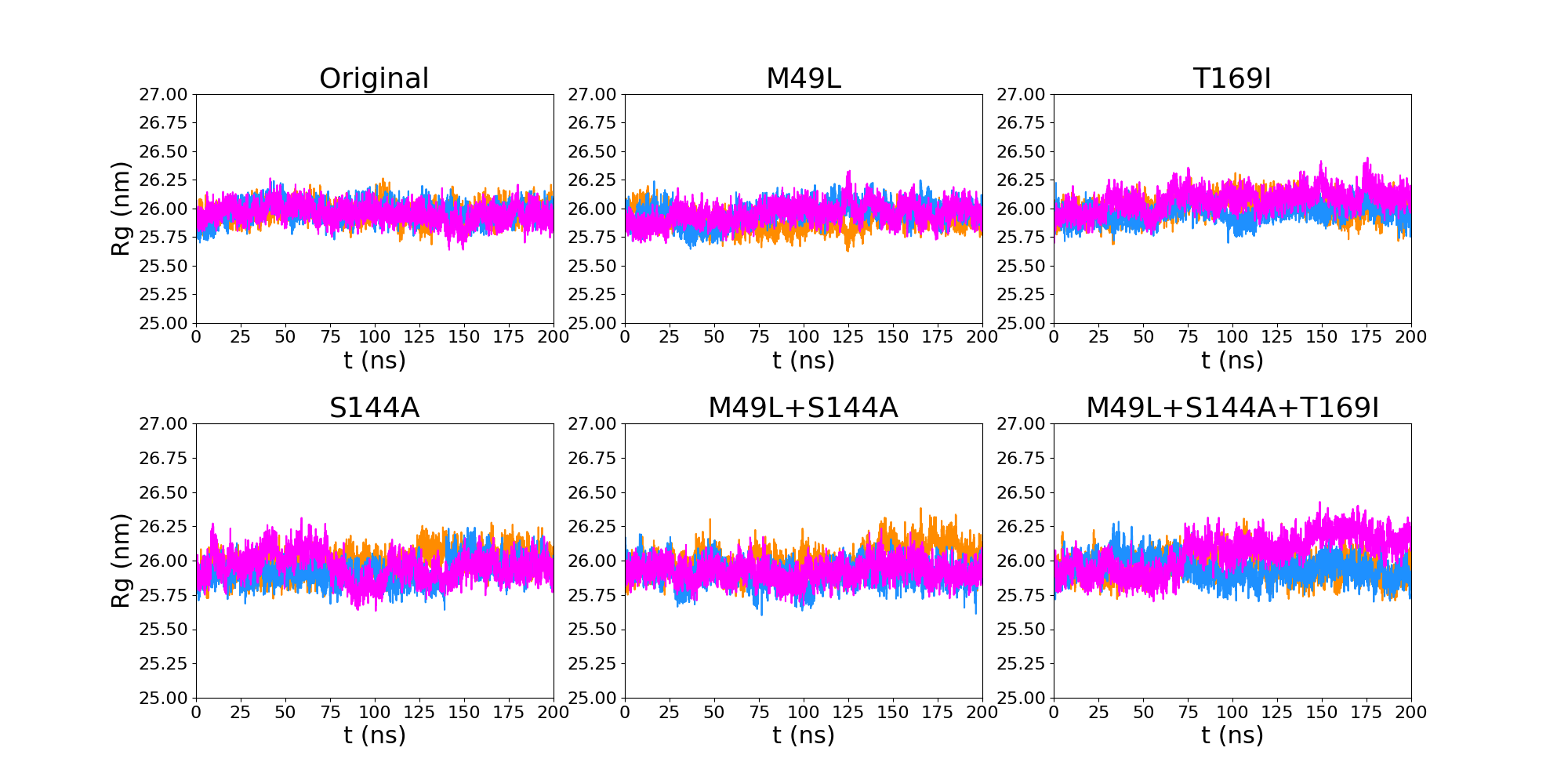


**Supplementary Figure S8. Time evolution of the radius of gyration (Rg) for Mpro-ensitrelvir complexes during molecular dynamics simulations.**

Rg values were calculated using all atoms from the individual structures corresponding to each of the three MDS. Top row, left to right: Original Mpro; M49L; T169I. Bottom row, left to right: S144A; M49L+S144A; M49L+S144A+T169I. The three MDS are represented in different colors.


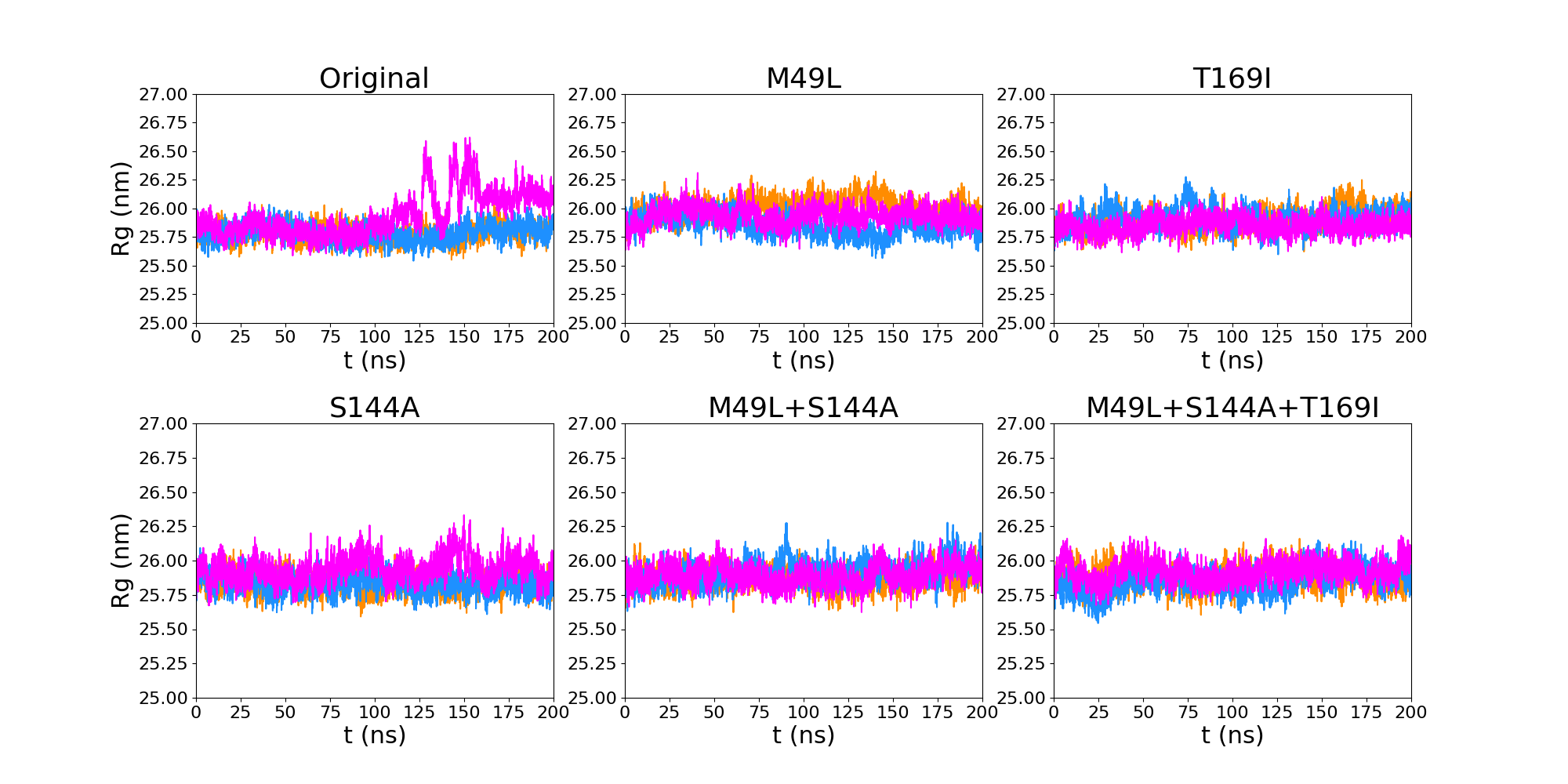


**Supplementary Figure S9. Time evolution of the radius of gyration (Rg) for Mpro-substrate peptide complexes during molecular dynamics simulations.**

Rg values were calculated using all atoms from the individual structure corresponding to each of the three MDS. Top row, left to right: Original Mpro; M49L; T169I. Bottom row, left to right: S144A; M49L+S144A; M49L+S144A+T169I. The three MDS are represented in different colors.


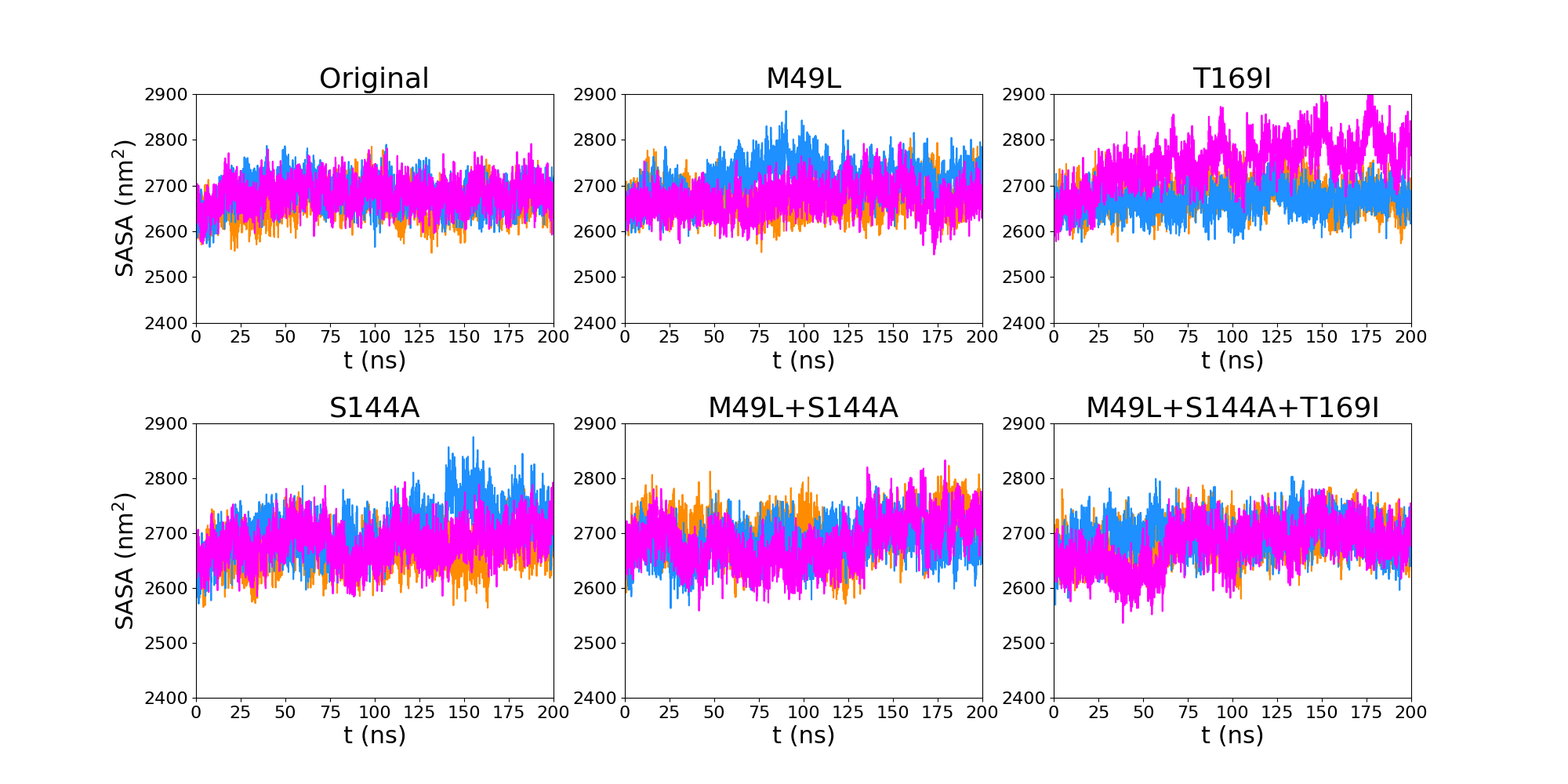


**Supplementary Figure S10. Time evolution of the solvent accessible surface areas (SASAs) for Mpro-ensitrelvir complexes during molecular dynamics simulations.**

SASAs were calculated using all atoms from the individual structures corresponding to each of the three MDS. Top row, left to right: Original Mpro; M49L; T169I. Bottom row, left to right: S144A; M49L+S144A; M49L+S144A+T169I. The three MDS are represented in different colors.


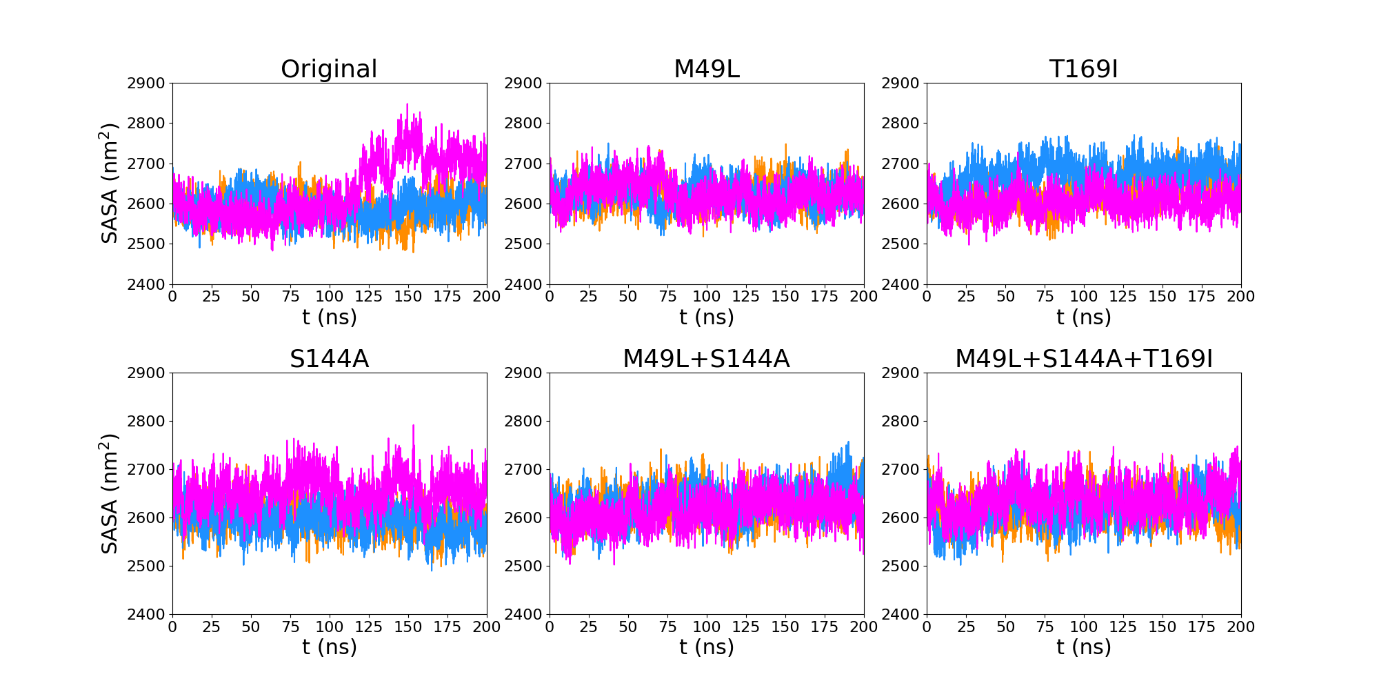


**Supplementary Figure S11. Time evolution of the solvent accessible surface areas (SASAs) for Mpro-substrate peptide complexes during molecular dynamics simulations.**

SASAs were calculated using all atoms from the individual structures corresponding to each of the three MDS. Top row, left to right: Original Mpro; M49L; T169I. Bottom row, left to right: S144A; M49L+S144A; M49L+S144A+T169I. The three MDS are represented in different colors.

**Supplementary Tables**

**Supplementary Table S1.** **Non-synonymous mutations in ensitrelvir escape viruses observed throughout the complete open reading frame (ORF) (compare Figure 1(A)).**

^a^Escape 1, which led to the polyclonal escape virus ENS-EV1, involved a primary escape culture followed by six viral passages in VeroE6 cells. The primary culture was treated with 4-fold EC50 ensitrelvir for 8 days. Subsequent passage (P) cultures were treated with increasing concentrations of ensitrelvir: The 1st passage (P1) culture with 8-fold EC50 for 3 days, the 2nd passage (P2) culture with 24-fold EC50 for 4 days, the 3rd passage (P3) culture with 81-fold EC50 for 6 days, the 4th passage (P4) culture with 162-fold EC50 for 3 days, the 5th passage (P5) culture with 324-fold EC50 for 6 days and the 6th passage (P6) culture with 364-fold EC50 for 6 days.

^b^%, frequency of non-synonymous nucleotide changes detected in the complete ORF by NGS. The analyzed viruses were collected from cell culture supernatants. A dash (-) indicates a frequency below 10%. Changes were included in the table that occurred at ≥10% in at least one viral culture.

^c^Escape 2, which led to the polyclonal escape virus ENS-EV2, involved a primary escape culture followed by four viral passages in VeroE6 cells. The primary culture was treated with 5-fold EC50 ensitrelvir for 13 days. Subsequent passage (P) cultures were treated with increasing concentrations of ensitrelvir: The 1st passage (P1) culture with 8-fold EC50 for 3 days), the 2nd passage (P2) culture with 81-fold EC50 for 6 days), the 3rd passage (P3) culture with 324-fold EC50 for 8 days and the 4th passage (P4) culture with 364-fold EC50 for 8 days.

^d^The nucleotide or amino acid residues given before the position numbers correspond to the original virus. The nucleotide or amino acid changes observed during passages under ensitrelvir treatment are shown after the position numbers.

^e^The SARS-CoV-2 protein associated with the identified change, corresponding to the original virus.

^f^Details of the conditions for viral genome sampling for NGS, specifying the fold EC50 applied, as well as the passage (P) and/or day (D) post-infection.

**Supplementary Table S2. Non-synonymous mutations in first passage virus stocks of ensitrelvir escape variants observed throughout the ORF (compare Figure 2(C)).**

^a^%, frequency of non-synonymous nucleotide changes detected in the complete ORF of the 1st passage virus stocks of recombinant SARS-CoV-2 variants, as analyzed by NGS. The analyzed viruses were collected from cell culture supernatants. A dash (-) indicates a frequency below 10%. Changes were included in the table that occurred at ≥10% in at least one viral culture.

^b^The nucleotide or amino acid residues given before the position numbers correspond to the original virus. The nucleotide or amino acid changes observed in first passage virus stocks are shown after the position numbers.

^c^The SARS-CoV-2 protein associated with the identified change, corresponding to the original virus.

^d^1st passage (P) virus stock.

**Supplementary Table S3. Non-synonymous mutations in fourth passage ensitrelvir escape variants observed throughout the ORF (compare Figure 2(C)).**

^a^%, frequency of non-synonymous nucleotide changes detected in the complete ORF of the recombinant SARS-CoV-2 variants following 4 viral passages, as analyzed by NGS. The analyzed viruses were collected from cell culture supernatants. A dash (-) indicates a frequency below 10%. Changes were included in the table that occurred at ≥10% in at least one viral culture.

^b^The nucleotide or amino acid residues given before the position numbers correspond to the original virus. The nucleotide or amino acid changes observed following four viral passages are shown after the position numbers.

^c^The SARS-CoV-2 protein associated with the identified change, corresponding to the original virus.

^d^Specification of the recombinant SARS-CoV-2 variants and NGS sampling time: viral genomes for variants were sampled at passage 4 (P4), day 2 (D2) or day 3 (D3) post-infection.

**Supplementary Table S4. Natural occurrence of substitutions in SARS-CoV-2 Mpro (compare Figure 2(D) and Figure 8(A)).**

^a^SARS-CoV-2 Mpro positions to which resistance associated substitutions investigated in this study localized.

^b^One-letter codes are used to represent amino acid residues; 'del' signifies a deletion.

^c^A total of 17,007,971 SARS-CoV-2 sequences were retrieved for analysis from the GISAID database on October 11, 2024.

^d^Total number of viruses harboring substitutions at positions 49, 144, 166, or 169 in Mpro.

**Supplementary Table S5. Frequency of naturally occurring substitutions at SARS-CoV-2 Mpro positon E166 (compare Figure 2(D) and Figure 8(A)).**

^a^%, frequency of amino acid changes at SARS-CoV-2 Mpro position E166 was analysed using the GISAID database on April 18th, 2022, and October 11, 2024. Values were rounded down to ten decimal places.

^b^One-letter codes are used to represent amino acid residues; 'del' signifies a deletion.

^c^Frequency changes were determined as Frequency (October, 2024)/Frequency (April, 2022) rounded down to the nearest tenth.

**Supplementary Table S6. Non-synonymous mutations in first passage virus stocks of E166 variants observed throughout the ORF (compare Figure 7(B)).**

^a^%, frequency of non-synonymous nucleotide changes detected in the complete ORF of the 1st passage virus stocks of recombinant SARS-CoV-2 variants with amino acid changes at Mpro-position 166, as analyzed by NGS. The analyzed viruses were collected from cell culture supernatants. A dash (-) indicates a frequency below 10%. Changes were included in the table that occurred at ≥10% in at least one viral culture.

^b^The nucleotide or amino acid residues given before the position numbers correspond to the original virus. The nucleotide or amino acid changes observed in first passage virus stocks are shown after the position numbers.

^c^The SARS-CoV-2 protein associated with the identified change, corresponding to the original virus.

^d^1st passage (P) virus stock.

**Supplementary Table S7. Non-synonymous mutations in fourth passage E166 variants observed throughout the ORF (compare Figure 7(B)).**

^a^%, frequency of non-synonymous nucleotide changes detected in the complete ORF of the recombinant SARS-CoV-2 variants with amino acid changes at Mpro-position 166 following 4 viral passages, as analyzed by NGS. The analyzed viruses were collected from cell culture supernatants. A dash (-) indicates a frequency below 10%. Changes were included in the table that occurred at ≥10% in at least one viral culture.

^b^The nucleotide or amino acid residues given before the position numbers correspond to the original virus. The nucleotide or amino acid changes observed following four viral passages are shown after the position numbers.

^c^The SARS-CoV-2 protein associated with the identified change, corresponding to the original virus.

^d^Specification of the recombinant SARS-CoV-2 variants and NGS sampling time: viral genomes for variants were sampled at passage 4 (P4), day 2 (D2) or day 3 (D3) post-infection.
